# Supplementary material for: Allosteric regulation and crystallographic fragment screening of SARS-CoV-2 NSP15 endoribonuclease
Source: Nucleic Acids Res. 2023 Apr 28;51(10):5255–70. doi: 10.1093/nar/gkad314 (PMC10250223; doi:10.1093/nar/gkad314)
Supplement: gkad314_Supplemental_Files [file gkad314_supplemental_files.zip › Supplementary datasets.zip!/160/Refine_8/01-BUSTER/Cycle-4/LIST.html]

Refining

**BUSTER 2.X**: A suite of programs for macromolecular structure refinement by maximum-likelihood and Bayesian methods  
Copyright © 1992-2020 Global Phasing Ltd.  
All rights reserved.  
Authors: G. Bricogne, E. Blanc, M. Brandl, C. Flensburg, P. Keller, W. Paciorek, P. Roversi, O. Smart, C. Vonrhein, T. Womack  

Please reference use of the software as:

- BUSTER-TNT 2.X, Global Phasing Ltd, Sheraton House, Cambridge CB3 0AX, UK

Version: 2.10.3 for linux64-ifort made on May 20 2020 at 01:14:34  
Licenced to: Diamond Light Source, Scientific Software  

```
---


### Parsing of the BUSTER cardfile

TITLE : autoBUSTER refinement

   Cell information

   Symmetry information

 Using CCP4 X-ray form factors.

   Missing structure chemical composition

 Using ATOM block for chemical composition.


   Shell information

### Parsing of the TNT cardfile


---


### Reading observations file

 Number of reflexions in input:  62372

      Number of accepted reflexions:  58271

      Number of rejected reflexions:      0

   Reflexions in test set:            2953

   Reflexions in working set:        55318


---


### 1. Generating initial model

 2.10 * Shannon sampling rate. NX NY NZ:   288  288  240


  Effective Shannon rates are:    2.22    2.22    2.55


   Allocated BUFFER of size:     76.465 Mb.

   Initial Geometry Information


 No missing atoms declared


   Summary of model volumes

 No missing atoms declared: no prior computed


 Computing bulk solvent model scattering


 NCSMASK PDB radius for solvent mask:     2.15 Angstrom

 running PDBSET (setting cell and spacegroup P1) ... done
 running NCSMASK [1] (creating mask, SMOOTH 1.04572) ... done
 running MAPDUMP (to check if reduction to unit cell is needed) ... done
 running MAPMASK (converting mask to map) ... done

   Generation of babslv by theta-filter

   Electron density in frg mask:  0.420 Å-1

 FOBS  put on rough absolute scale by Wilson plot :
           K        =  0.67039E+02
           B        =     -9.809

 

### Cycle 0001: MaxLik scaling and refinement


   MaxLik Scaling Cycle 0001

   Wilson plot

   Statistics in resolution bins just after scaling
```

|  |  |
| --- | --- |
|  | - Rxpct factors plot- Rxpct factors table- <Log-Lik Gain>- Ln Sigma A- Correlation Coefficients |

```
   <Log-Likelihood> of null-hypothesis

Structure Factor Amplitudes


   Average Figure of Merit

 Switching off refinement of overall temperature factor


 Added B =     0.0263 Å2 to all atoms in fragment


 Added B =     0.0263 Å2 to B_SOLV

```
gelly refinement module
 Authors (2004-2009) O. S. Smart, C. Flensburg, W. Paciorek, C. Vonrhein, 
                     M. Brandl, T. O. Womack and G. Bricogne
         (2010-2011) O. S. Smart, C. Flensburg, W. Paciorek, C. Vonrhein, 
                     T. O. Womack and G. Bricogne
         (2012-2014) O. S. Smart, C. Flensburg, W. Paciorek, C. Vonrhein, 
                     and G. Bricogne
         (2015-2020) G. Bricogne, C. Flensburg, W. Paciorek, C. Vonrhein
 Copyright © 2004-2020 Global Phasing Ltd. 
 All rights reserved.
 gelly module compilation date May 20 2020 01:11:20
 
 gelly uses routines from TNT geometry. References: 
     Tronrud, D. E., Ten Eyck, L. F., Matthews, B. W. (1987)
     "An Efficient General-Purpose Least-Squares Refinement Program 
     for Macromolecular Structures". Acta Crystallogr A, 43:489-501.
     Tronrud, D. E. (1997). "The TNT Refinement Package", 
     in Macromolecular Crystallography, Part B, Eds Charlie Carter 
     and Robert Sweet, Methods in Enzymology, 277:306-319.
 wildcard matching function by Julian Robichaux http://www.nsftools.com

 No -random_seed flag found. Random number stream initialised using:
2147483562
2147483398

 Control flags set to:  -autoncs -hydrogens_input_occ2zero \
                     -hydrogens_output_occ2heavy -screen \
                     100 -screen_sigma \
                     3.0 -keeppdb \
                     1000000 -taomonitor_verbose \
                     2 
 

 ############### Starting setup via TNT routines

 Setting up tnt routines including license check:

                   TNT Stereochemistry Program
 Version: 5.22.3.0
 Licence for Diamond Light Source, Scientific Software is valid
 
 Using TNT geometry routines to load the tnt control file: gelly.tnt
 Using TNT routines to load initial coordinates from file fragment.cor
 Expecting 6503 atoms

 Found     2188 "NOTE BUSTER_RESIDUE_ATOM_DATA" cards
 Have copied information for    6503 atoms from TNT to gelly's store 
 
 The TNT WEIGHT cards used by this run:
 -----WEIGHT       BOND     2.0
 -----WEIGHT       ANGLE    2.0
 -----WEIGHT       IMPROPER 0.0
 -----WEIGHT       TORSION  2.0
 -----WEIGHT       PSEUDO   0.0
 -----WEIGHT       TRIGONAL 2.0
 -----WEIGHT       PLANE    5.0
 -----WEIGHT       CONTACT  5.0
 -----WEIGHT       BCORREL  10.0
 -----WEIGHT       CHIRAL   5.0
 -----WEIGHT       NCS      50.0
 -----WEIGHT       IDEAL    4.0
 -----WEIGHT       RFACTOR  7.54
 
 Dictionary versions picked up from NOTE BUSTER_RESTRAINT_DICT cards as: 
 ------ protgeo_eh99.dat (V1.6.4.17) 20191001  STANDARD AMINO ACID DICTIONARY. BONDS AND ANGLES FROM ENGH AND HUBER EH99. OTHER VALUES BASED ON PREVIOUS TNT OR TAKEN FROM CCP4. INCLUDES HYDROGEN ATOMS.
 ------ exoticaa.dat (V1.3.4.15) 20200327  COLLECTION OF NON-STANDARD AMINO ACIDS, MAINLY EH91 WITHOUT IDEAL DISTANCE INFO
 ------ nuclgeo.dat (V1.13.4.8) 20190909
 ------ bcorrel.dat (V1.15.10.2) 20190616
 ------ contact.dat (V1.15.12.10) 20191121
 ------ idealdist_contact.dat (V1.3.4.12) 20191001  IDEAL-DISTANCE CONTACT TERM DATA AS USED IN PROLSQ. VALUES USED HERE ARE BASED ON DATA HARVESTING OF PDB AS OF 20190109.
 ------ restraints for LIG (Unknown) from cif dictionary Z1636723439.cif
 ------ restraints for CIT (CITRIC ACID) from cif dictionary CIT.cif; generated by GRADE_PDB_LIGAND 1.2.12 (October 4 2016) using MOGUL 1.7.2(RC1), CSD as538be, with quantum mechanics RM1 ((DatabaseID PDB-CIT))
 ------ assume.dat (V1.9.4.2) 20180705
 
 Have picked up spacegroup name for pdb output as 'P 63      '
 Setting up Chain/Residue arrays
 
 Have command line specification -autoncs*
     Have found       1 NCS relations
     Pairwise matching residue number and type between chains: 
                    A        B        S        W    
          A         .       99%       -        -   
          B                  .        -        -   
          S                           .        -   
          W                                    .   
       Table key--                                                       
          all   : two chains have 100% match and same number of residues 
         multi  : ditto and they contribute to a multi chain ncs cluster 
          ***%  : two chains have >80% matching residue number/type      
           -    : chains appear to be unrelated                          
      chain A     have identified ncs to     1 other chains. So will weight each autoncs by (       6.666/#)=      6.6660
      chain B     have identified ncs to     1 other chains. So will weight each autoncs by (       6.666/#)=      6.6660
      chain S     have identified ncs to     0 other chains. 
      chain W     have identified ncs to     0 other chains. 
     For .Gelly cards produced see below - after pruning
 
 Setting up BUSTER_SET groups
 Have found    89 BUSTER_SET cards
 Now silently applying automatic/dictionary set definitions. For a verbose listing use '-verbose_set' flag.
 Applying user defined set definitions:
 =====NOTE BUSTER_SET ncsautoXcld = Water --> set 'ncsautoXcld' now has   966 atoms 
 =====NOTE BUSTER_SET ncsautoABset = Chain_A \ ncsautoXcld --> set 'ncsautoABset' now has  2770 atoms 
 Have defined    49 sets: 
      set:                 'All' has   6503 atoms in   1666 residues - automatically defined
      set:               'Empty' has      0 atoms in      0 residues - automatically defined
      set:            'ExoticAA' has      0 atoms in      0 residues - defined by dictionary file
      set:           'ExoticNUC' has      0 atoms in      0 residues - defined by dictionary file
      set:              'NotHET' has   5514 atoms in    698 residues - defined by dictionary file
      set:             'Chain_A' has   2770 atoms in    350 residues - automatically defined
      set:             'Chain_B' has   2767 atoms in    350 residues - automatically defined
      set:             'Chain_S' has    567 atoms in    567 residues - automatically defined
      set:             'Chain_W' has    399 atoms in    399 residues - automatically defined
      set:               'Water' has    966 atoms in    966 residues - defined by dictionary file
      set:          'StdProtein' has   5514 atoms in    698 residues - defined by dictionary file
      set:             'Protein' has   5514 atoms in    698 residues - defined by dictionary file
      set:                'Back' has   2794 atoms in    698 residues - defined by dictionary file
      set:                'Side' has   2720 atoms in    650 residues - defined by dictionary file
      set:        'RnaCanonical' has      0 atoms in      0 residues - defined by dictionary file
      set:        'DnaCanonical' has      0 atoms in      0 residues - defined by dictionary file
      set:     'RnaDnaCanonical' has      0 atoms in      0 residues - defined by dictionary file
      set:     'DnaRnaCanonical' has      0 atoms in      0 residues - defined by dictionary file
      set:         'RnaExtended' has      0 atoms in      0 residues - defined by dictionary file
      set:         'DnaExtended' has      0 atoms in      0 residues - defined by dictionary file
      set:      'RnaDnaExtended' has      0 atoms in      0 residues - defined by dictionary file
      set:      'DnaRnaExtended' has      0 atoms in      0 residues - defined by dictionary file
      set:                 'Dna' has      0 atoms in      0 residues - defined by dictionary file
      set:                 'Rna' has      0 atoms in      0 residues - defined by dictionary file
      set:              'RnaDna' has      0 atoms in      0 residues - defined by dictionary file
      set:              'DnaRna' has      0 atoms in      0 residues - defined by dictionary file
      set:             'Nucleic' has      0 atoms in      0 residues - defined by dictionary file
      set:             'Polymer' has   5514 atoms in    698 residues - defined by dictionary file
      set: 'BufferWeichenberger' has     13 atoms in      1 residues - defined by dictionary file
      set:    'BufferAdditional' has      0 atoms in      0 residues - defined by dictionary file
      set:              'Buffer' has     13 atoms in      1 residues - defined by dictionary file
      set:         'MetalAlkali' has      0 atoms in      0 residues - defined by dictionary file
      set:  'MetalAlkalineEarth' has      0 atoms in      0 residues - defined by dictionary file
      set:     'MetalTransition' has      0 atoms in      0 residues - defined by dictionary file
      set: 'MetalPostTransition' has      0 atoms in      0 residues - defined by dictionary file
      set:    'MetalLanthanides' has      0 atoms in      0 residues - defined by dictionary file
      set:               'Metal' has      0 atoms in      0 residues - defined by dictionary file
      set:         'SugarAlphaD' has      0 atoms in      0 residues - defined by dictionary file
      set:         'SugarAlphaL' has      0 atoms in      0 residues - defined by dictionary file
      set:               'Sugar' has      0 atoms in      0 residues - defined by dictionary file
      set:               'Other' has     23 atoms in      2 residues - defined by dictionary file
      set:            'Hydrogen' has      0 atoms in      0 residues - defined by dictionary file
      set:         'nonHydrogen' has   6503 atoms in   1666 residues - defined by dictionary file
      set:             'OccZero' has      0 atoms in      0 residues - defined by dictionary file
      set:          'OccNonZero' has   6503 atoms in   1666 residues - defined by dictionary file
      set:             'OccFull' has   6461 atoms in   1663 residues - defined by dictionary file
      set:          'OccNonFull' has     42 atoms in      3 residues - defined by dictionary file
      set:         'ncsautoXcld' has    966 atoms in    966 residues - user specified
      set:        'ncsautoABset' has   2770 atoms in    350 residues - user specified
 No command line arguments '-occ_allhet' or '-occ_restyp' so will not setup automated occcupancy refinement
 
 Interpreting CONSTANT and FREE cards: 
 =====CONSTANT     OCC
 Have found and interpreted      1 CONSTANT cards in the TNT input. 
 Before any COMBINE - Number of atoms with fixed XYZ =     0
 Before any COMBINE - Number of atoms with fixed B   =     0
 Before any COMBINE - Number of atoms with fixed OCC =  6503
 Before any COMBINE - Number of atoms with fixed BIJ =   966
                            cf total number of atoms =  6503
 
 There are no COMBINE cards to be found in the TNT card input 
 
 There are no NOTE BUSTER_COMBINE_RES cards to be found in the TNT card input 

 ############### Working out full connectivity list


 ############### Working out equivalent atoms


 Residue with most atoms given below:
        A|58:N(TRP)         
        A|58:CA(TRP)        
        A|58:CB(TRP)        
        A|58:CG(TRP)        
        A|58:CD1(TRP)       
        A|58:NE1(TRP)       
        A|58:CE2(TRP)       
        A|58:CD2(TRP)       
        A|58:CE3(TRP)       
        A|58:CZ3(TRP)       
        A|58:CH2(TRP)       
        A|58:CZ2(TRP)       
        A|58:C(TRP)         
        A|58:O(TRP)         


 Have no '-equiv_check_plus' argument - so will take atom type into
 account when checking for equivalence of otherwise identical atoms.
 

 ############### Looking at dihedrals


 ############### Looking at chirals

 NOTE BUSTER_CHIRAL_ANGLECUT card found - read value=    -31.00 degs from it
 NOTE BUSTER_CHIRAL_SIGMA card found - read value=      1.70 degs from it
 RANGE allowed for B variables in refinement:   from      3.00000 to    300.00000

 ############### Preparing for PDB output


 ############### Processing extra restraints explicitly defined

 No 'NOTE BUSTER_TARGET' cards or -target?? args found (used to read coordinates files for soft ncs to external)
 No 'NOTE BUSTER_OCCSUM'     cards found - so not using sum of occupancy restraints
 No 'NOTE BUSTER_DISTANCE'   cards found - so not setting up any additional atom-atom distance restraints
 No 'NOTE BUSTER_UTILANGLE'  cards found - so not setting up any additional restraints on any angle
 No 'NOTE BUSTER_UTILTOR'    cards found - so not setting up any additional sinusoidal restraints on torsions
 
 --- Have some similarity definitions 
     Similarity restraints on RMSD use superposition routines from: 
     Coutsias, E.A., Seok, C., Dill, K.A.(2004)
     "Using quaternions to calculate RMSD", J. Comput. Chem., 25:1849-1857 

 Have found    1 'NOTE BUSTER_SIM_DEFINE' cards 
     Maximum number of chains involved in any SIM group (excluding the template)=     1


 ########## Interpreting card: NOTE BUSTER_SIM_DEFINE ncsautoAB ncsautoABset B
     Number of atoms in template set=  2770
     Number of related chains (other than template) in SIM set=    1
     List of chains to be restrained (excluding template)=   B   
     Pairwise RMSD, XYZ in Angstroms, for SIM group: ncsautoAB
                 A         B   
     A         -----     ----- 
     B         0.601     ----- 
     Overall RMSD,XYZ for the SIM group is     0.601 Angstroms
     Pairwise rms deviation of [individual B's minus their set/chain average] in Angs**2
                 A         B         Bavg
     A         -----     -----     77.18
     B         18.47     -----     64.99
     Overall RMSD,B   for the SIM group is    18.475 Angs**2
     The number of common atoms for the above fits:
                 A         B   
     A         -----     ----- 
     B          2757     ----- 
     (compared to the template set that has    2770 atoms in it)
     List of atoms that are absent in one or more chains for this group: 
                                    A    B   
                    C1      401     +    -
                    O1      401     +    -
                    O2      401     +    -
                    C2      401     +    -
                    C3      401     +    -
                    O7      401     +    -
                    C4      401     +    -
                    C5      401     +    -
                    O3      401     +    -
                    O4      401     +    -
                    C6      401     +    -
                    O5      401     +    -
                    O6      401     +    -
     (key  +  atom present for this chain 
           -  atom absent  for this chain)
 transforms for each of the superpositions orthogonal matrix r11 r12 r13 r21 r22 r23 r31 r32 r33 tx ty tz (PDBSET syntax): 
TRANS_AB -0.877985 -0.478504 -0.013270 -0.478565  0.878050  0.001705  0.010836  0.007848 -0.999910    33.940    32.942    15.248
 reverse transforms 
TRANS_BA -0.877985 -0.478565  0.010836 -0.478504  0.878050  0.007848 -0.013270  0.001705 -0.999910    45.398   -12.804    15.640
 
 Setting up LSSR interatomic distance restraints
 Interpreting card: NOTE BUSTER_SIM_RESTRAIN_LSSR ncsautoAB 6.666
        setting up interatomic distance pair restraints with:
        Dmin=         0.00
        Dmax=         5.50
        sigma=        0.20
        Vmax=         3.00
        weight=       6.67
        number of distance pairs defined for this set is      34865
 Have not supplied either '-sim_swap_equiv' or '-sim_swap_equiv_plus' argument 
   so will not try swaping equivalent atoms to improve NCS and/or target agreement.
 
 NCS by LSSR outlier analysis
 -autoncs option automatic NCS prune routine -------- 
 Residues pruned out by gradient condition:
           A|34     A|135     A|137     A|147     A|148     A|150     A|151
          A|173     A|181     A|184     A|185     A|186     A|187     A|189
          A|216     A|217     A|220     A|234     A|235     A|236     A|238
          A|239     A|240     A|244     A|245     A|246     A|247     A|248
          A|249     A|250     A|253     A|256     A|280     A|281     A|287
          A|288     A|289     A|292     A|293     A|294     A|295     A|317
          A|318     A|319     A|327     A|328     A|329     A|343     A|344
          A|345      B|34     B|135     B|137     B|147     B|148     B|150
          B|151     B|173     B|181     B|184     B|185     B|186     B|187
          B|189     B|216     B|217     B|220     B|234     B|235     B|236
          B|238     B|239     B|240     B|244     B|245     B|246     B|247
          B|248     B|249     B|250     B|253     B|256     B|280     B|281
          B|287     B|288     B|289     B|292     B|293     B|294     B|295
          B|317     B|318     B|319     B|327     B|328     B|329     B|343
          B|344     B|345
  
 Residues pruned out by function/max condition:
          A|149     A|187!    A|188     A|220!    A|259     A|319!    A|346 
         A|ENDA     B|149     B|187!    B|188     B|220!    B|259     B|319!
          B|346    B|ENDA 
  
 Total number of residues for which LSSR restraints have been pruned out is      110 out of      698 participating
 Number of LSSR restraints pruned out is      7502 this is   21.5% of the total           34865
  
 -autoncs .Gelly cards suitable for adaptation for manual definition/pruning
           (note auto pruning is done in a slightly different way so exact results may differ slightly)
 -autoncs option .Gelly cards also written to file  auto_gelly_cards.txt
NOTE BUSTER_SET ncsautoXcld = Water    # card added - produced by -autoncs
NOTE BUSTER_SET ncsautoXcld = ncsautoXcld + { A|34 A|135 A|137 A|147 A|148 }    # card added - produced by -autoncs
NOTE BUSTER_SET ncsautoXcld = ncsautoXcld + { A|149 A|150 A|151 A|173 A|181 }    # card added - produced by -autoncs
NOTE BUSTER_SET ncsautoXcld = ncsautoXcld + { A|184 A|185 A|186 A|187 A|188 }    # card added - produced by -autoncs
NOTE BUSTER_SET ncsautoXcld = ncsautoXcld + { A|189 A|216 A|217 A|220 A|234 }    # card added - produced by -autoncs
NOTE BUSTER_SET ncsautoXcld = ncsautoXcld + { A|235 A|236 A|238 A|239 A|240 }    # card added - produced by -autoncs
NOTE BUSTER_SET ncsautoXcld = ncsautoXcld + { A|244 A|245 A|246 A|247 A|248 }    # card added - produced by -autoncs
NOTE BUSTER_SET ncsautoXcld = ncsautoXcld + { A|249 A|250 A|253 A|256 A|259 }    # card added - produced by -autoncs
NOTE BUSTER_SET ncsautoXcld = ncsautoXcld + { A|280 A|281 A|287 A|288 A|289 }    # card added - produced by -autoncs
NOTE BUSTER_SET ncsautoXcld = ncsautoXcld + { A|292 A|293 A|294 A|295 A|317 }    # card added - produced by -autoncs
NOTE BUSTER_SET ncsautoXcld = ncsautoXcld + { A|318 A|319 A|327 A|328 A|329 }    # card added - produced by -autoncs
NOTE BUSTER_SET ncsautoXcld = ncsautoXcld + { A|343 A|344 A|345 A|346 A|ENDA }    # card added - produced by -autoncs
NOTE BUSTER_SET ncsautoABset = Chain_A \ ncsautoXcld    # card added - produced by -autoncs
NOTE BUSTER_SIM_DEFINE ncsautoAB ncsautoABset B    # card added - produced by -autoncs
NOTE BUSTER_SIM_RESTRAIN_LSSR ncsautoAB 6.666    # card added - produced by -autoncs

 LSSR function contribution before pruning     50425.5
                             after pruning     10376.0
 Graph of which residues pruned from LSSR: ncs_pruned.mtv
 Graph of LSSR function outliers ncs_function_outliers.mtv
 Graph of LSSR gradient outliers ncs_gradient_outliers.mtv
 LSSR - analysis of distance difference distribution for NCS
         DeltaD less than 0.5 Angs:  proportion of population= 98.57% average DeltaD of subset     0.013 Angs
 Histogram of LSSR deltaD distribution ncs_histogram.mtv
 
 Interpreting card: NOTE BUSTER_TLS_SET tls1 { A|* }
 Interpreting card: NOTE BUSTER_TLS_SET tls2 { B|* }
 Interpreting card: NOTE BUSTER_TLS_SET tls1 { A|* }
 Interpreting card: NOTE BUSTER_TLS_T   tls1     -1.78214E-01 -9.18952E-02  2.39945E-01 -3.34788E-02  3.82832E-02  7.05171E-03
 Interpreting card: NOTE BUSTER_TLS_L   tls1      1.61533E+00  1.36273E+00  7.21546E-01  2.44049E-01 -5.13123E-01  3.00651E-01
 Interpreting card: NOTE BUSTER_TLS_S   tls1      2.26938E-01 -2.36880E-01  2.82462E-01 -1.82387E-01 -6.13010E-02 -2.07300E-01 -1.26450E-02 -1.05032E-01
 Interpreting card: NOTE BUSTER_TLS_O   tls1     -2.33533E+01 -8.36748E+01  1.13031E+01
 Interpreting card: NOTE BUSTER_TLS_SET tls2 { B|* }
 Interpreting card: NOTE BUSTER_TLS_T   tls2     -2.55147E-01 -1.97931E-01  3.33849E-01  1.93375E-02  4.44013E-02 -1.38678E-02
 Interpreting card: NOTE BUSTER_TLS_L   tls2      1.50139E+00  1.37402E+00  9.53074E-01  7.53471E-01 -8.67385E-01 -7.93362E-01
 Interpreting card: NOTE BUSTER_TLS_S   tls2     -5.06241E-02  1.57153E-01 -1.23636E-01  1.18337E-01  2.46232E-01  7.25730E-02 -9.77238E-02  6.96725E-02
 Interpreting card: NOTE BUSTER_TLS_O   tls2      2.20712E+01 -7.19054E+01  3.51200E+00
 Interpreting card: NOTE BUSTER_TLS_FIX tls1 ALL
 Interpreting card: NOTE BUSTER_TLS_FIX tls2 ALL


 TLS information. Total number of groups:     2


 Group   Natoms    FixRB   FixTLS   Group Tag    Group Spec
     1     2770      Y        Y     tls1    { A|* }
     2     2767      Y        Y     tls2    { B|* }
 
NOTE BUSTER_TLS_SET tls1 { A|* }
NOTE BUSTER_TLS_T   tls1     -1.78214E-01 -9.18952E-02  2.39945E-01 -3.34788E-02  3.82832E-02  7.05171E-03
NOTE BUSTER_TLS_L   tls1      1.61533E+00  1.36273E+00  7.21546E-01  2.44049E-01 -5.13123E-01  3.00651E-01
NOTE BUSTER_TLS_S   tls1      2.26938E-01 -2.36880E-01  2.82462E-01 -1.82387E-01 -6.13010E-02 -2.07300E-01 -1.26450E-02 -1.05032E-01
NOTE BUSTER_TLS_O   tls1     -2.33533E+01 -8.36748E+01  1.13031E+01
NOTE BUSTER_TLS_FIX tls1 ALL
 
NOTE BUSTER_TLS_SET tls2 { B|* }
NOTE BUSTER_TLS_T   tls2     -2.55147E-01 -1.97931E-01  3.33849E-01  1.93375E-02  4.44013E-02 -1.38678E-02
NOTE BUSTER_TLS_L   tls2      1.50139E+00  1.37402E+00  9.53074E-01  7.53471E-01 -8.67385E-01 -7.93362E-01
NOTE BUSTER_TLS_S   tls2     -5.06241E-02  1.57153E-01 -1.23636E-01  1.18337E-01  2.46232E-01  7.25730E-02 -9.77238E-02  6.96725E-02
NOTE BUSTER_TLS_O   tls2      2.20712E+01 -7.19054E+01  3.51200E+00
NOTE BUSTER_TLS_FIX tls2 ALL


 TLS: mmCIF group specification begin


#
loop_
_pdbx_refine_tls_group.id
_pdbx_refine_tls_group.refine_tls_id
_pdbx_refine_tls_group.beg_PDB_ins_code
_pdbx_refine_tls_group.beg_auth_asym_id
_pdbx_refine_tls_group.beg_auth_seq_id
_pdbx_refine_tls_group.end_PDB_ins_code
_pdbx_refine_tls_group.end_auth_asym_id
_pdbx_refine_tls_group.end_auth_seq_id
_pdbx_refine_tls_group.selection
_pdbx_refine_tls_group.selection_details
   1   1 . A   -1 . A  346 . '{ A|* }'
   2   1 . A  401 . A  401 . '{ A|* }'
   3   2 . B   -1 . B  346 . '{ B|* }'
   4   2 . B  401 . B  401 . '{ B|* }'
#


 TLS: mmCIF group specification end


 Interpreting card: NOTE BUSTER_ANISO_SET Empty
 Interpreting card: NOTE BUSTER_ANISO_WEIGHT SCA  16.0
 Interpreting card: NOTE BUSTER_ANISO_WEIGHT SPH   0.6    0.6
 Interpreting card: NOTE BUSTER_ANISO_WEIGHT SIM   7.8    4.8    2.6    0
 Interpreting card: NOTE BUSTER_ANISO_WEIGHT RIG  21.5   21.5    3.7    0


 After TLS and ANISO: Number of atoms with fixed/combined xyz =     0
 After TLS and ANISO: Number of atoms with fixed/combined B   =     0
 After TLS and ANISO: Number of atoms with fixed/combined OCC =  6503
 After TLS and ANISO: Number of atoms with fixed/combined BIJ =  6503
 After TLS and ANISO: Number of atoms in a TLS group          =  5537
                                     cf total number of atoms =  6503
 

 ############### Setup state vector

 No free_vect_ulim_xyz argument found: using default of  100.000
 No free_vect_llim_xyz argument found: using default of -100.000
 Number of parameters added for TLS description                 =       0
 Number of parameters to describe molecular conformation        =   26012

 ############### Setup free vector

 Number of free parameters to be adjusted in refinement   =   26012

 ############### Setting up own geometry


 No command line argument '-tnt_fn' so will use gelly to do geometry function calculations

 ============= Call TNT functions


 ============= Get TNT symmetry information

 
      Getting symmetry operators from TNT. 
          gelly will classify symmetry using pdb-like convention: 
               SYMOP   SYMMETRY
              NNNMMM   OPERATOR
                1555   X,Y,Z
                2555   -Y,X-Y,Z
                3555   -X+Y,-X,Z
                4555   -X,-Y,1/2+Z
                5555   Y,-X+Y,1/2+Z
                6555   X-Y,X,1/2+Z
          where NNN -> operator number and MMM -> translation vector
 

 ============= Own Gelly geometry/restraints setup


 ----------- Identify bond-types


    Setting up GEOMBONDREC_TYPE_HASH* to be able to map a given bond type (e.g.
    "single") to an internal integer (GBOND_TYPE=1) etc.

    Using default  GEOMBONDREC_TYPE_HASH_STR='SING* 1 DOUB* 2 TRIP* 3 AROM* 4 DELO* 4 METAL* 5':
    to override default use a NOTE BUSTER_GEOMBONDREC_TYPE_HASH_STR gelly card.

 ----------- Classify torsion into peptide omega and other


 ----------- Setup of non-bonded terms

 Ideal-distance contact term setup:
    Restraint function constants (normally these values are from NOTE BUSTER_IDEAL_CONTACT_* cards)
    Sigma for general contacts      0.200 Angs
    Sigma for 1-4 contacts          0.100 Angs (contacts across a torsion)
    Sigma for 1-5 contacts          0.150 Angs
    Sigma for 1-N contacts          0.200 Angs
    Sigma for HBOND pairs           0.100 Angs (contacts between HB_types A and D or B's)
    Contact distance increment for each atom in HBOND          -0.150 Angs 
    Contact distance increment for H-bond acceptor              0.100 Angs 
    Contact distance increment for 1-4 with an O atom          -0.050 Angs 
    Contact distance increment for 1-4 with an N atom          -0.180 Angs 
    Contact distance increment for 1-4 with an C atom          -0.370 Angs 
    Contact distance increment for 1-4 with an S atom          -0.150 Angs 
    Contact distance increment for 1-4 with an P atom          -0.150 Angs 
    Contact distance increment for 1-4 with any other atom     -0.150 Angs 
    All atoms matched for CCP4 atom type.
    Number of CCP4 atom types used is         24 (H-bound and not H-bound counted separately)

 Setting up short/ideal-distance contacts
   Using gelly special position handling. (For TNT instead rerun with argument '-special_tnt')

   No command line argument '-special_all' specified. We will auto-generate
   EXCLUDE cards only for atom-atom contacts across symmetry (i.e. on special
   position) when this involves a contact within a residue to itself.

   Have set up non-bonded exclusions - 1-3 contacts
   Atom A|27:CA         makes the most 1-3 contacts:  6

   Have set up non-bonded exclusions - 1-4 contacts
   Atom A|27:N          makes the most 1-4 contacts:  7

   Have set up non-bonded exclusions - 1-5 contacts
   Atom A|28:C.A        makes the most 1-5 contacts: 10

 ############################################################################################################
 ###################################################### setting up contact terms
 ############################################################################################################


 ============= Call Gelly versions of TNT functions

 WARNING: 1-4 contact without 1-(2,3)-4 data for A|401:O2(CIT)        and A|401:C3(CIT)        X-X
 WARNING: 1-4 contact without 1-(2,3)-4 data for A|401:C2(CIT)        and A|401:O6(CIT)        X-X
 WARNING: 1-4 contact without 1-(2,3)-4 data for A|401:O7(CIT)        and A|401:O5(CIT)        X-X
 WARNING: 1-4 contact without 1-(2,3)-4 data for A|401:C4(CIT)        and A|401:O6(CIT)        X-X
 WARNING: 1-4 contact without 1-(2,3)-4 data for B|401:C05(LIG)       and B|401:C08(LIG)       X-X
 WARNING: 1-4 contact without 1-(2,3)-4 data for B|401:C06(LIG)       and B|401:C09(LIG)       X-X

       TERM      weight      number      TNT_fn    Gelly_fn        diff
       BOND       2.000        5652    1863.714    1863.714       0.000
       ANGL       2.000        7669    5709.956    5709.956       0.000
       IMPR       0.000           0       0.000       0.000       0.000
       TORS       2.000        1967    3415.846    3415.847      -0.000
       TRIG       2.000           0       0.000       0.000       0.000
       PLAN       5.000         963    2727.769    2727.769       0.000
       CONT       5.000           0     158.667       0.000     158.667
       BCOR      10.000        5630    6195.272    8408.017   -2212.744


 ============= Call Gelly geometry functions

   Switching torsion functional form to sinusoidal rather than split harmonic functional.
      if you want this not to happen then rerun with '-torsharm'
      Weight for sinusoidal torsion=     2.000
   BCOR function/gradient will be split between H and non-H atoms (as for any atom pair). Use '-bcorrel_all_in_hydrogens' to change this.

 ----------- Fix B-correlation restraints

 
 Checking for bonds that do not have a bcorrel restraint defined
 We will wipe all existing BCORrels if OCC=0.0 atom(s) is/are involved. This can be' switched off via the '-bcorrel_nowipe_zeroocc' flag.
 We will wipe all existing BCORrels if only non-hydrogen atom(s) with fixed B(iso) are involved. This can be switched off via the '-bcorrel_nowipe_fixb_nonhydrogens' flag.
 We will keep all existing BCORrels if hydrogen atom(s) with fixed B(iso) is/are involved. This can be switched off via the '-bcorrel_wipe_fixb_hydrogens' flag.
 No -bcorrel_missing_sigma found. So will set sigma to     2.5 Ang**2 for any bond with no existing BCORrel
 No -bcorrel_missing_sigma_hydrogens found. So will set sigma to     1.0 Ang**2 for any bond with no existing BCORrel involving hydrogens

 No active BCORels found involving OCC=0.0 atom(s)


 No active BCORels found involving fixed-B (only) non-hydrogen atom(s)

    add a default BCOR for bond A|401:C1(CIT) A|401:C2(CIT)
    add a default BCOR for bond A|401:C1(CIT) A|401:O1(CIT)
    add a default BCOR for bond A|401:C1(CIT) A|401:O2(CIT)
    add a default BCOR for bond A|401:C2(CIT) A|401:C3(CIT)
    add a default BCOR for bond A|401:C3(CIT) A|401:C4(CIT)
    add a default BCOR for bond A|401:C3(CIT) A|401:C6(CIT)
    add a default BCOR for bond A|401:C3(CIT) A|401:O7(CIT)
    add a default BCOR for bond A|401:C4(CIT) A|401:C5(CIT)
    add a default BCOR for bond A|401:C5(CIT) A|401:O3(CIT)
    add a default BCOR for bond A|401:C5(CIT) A|401:O4(CIT)
    add a default BCOR for bond A|401:C6(CIT) A|401:O5(CIT)
    add a default BCOR for bond A|401:C6(CIT) A|401:O6(CIT)
    add a default BCOR for bond B|401:C01(LIG) B|401:N02(LIG)
    add a default BCOR for bond B|401:N02(LIG) B|401:C03(LIG)
    add a default BCOR for bond B|401:C03(LIG) B|401:O04(LIG)
    add a default BCOR for bond B|401:C03(LIG) B|401:C05(LIG)
    add a default BCOR for bond B|401:C05(LIG) B|401:C06(LIG)
    add a default BCOR for bond B|401:C05(LIG) B|401:O10(LIG)
    add a default BCOR for bond B|401:C06(LIG) B|401:N07(LIG)
    add a default BCOR for bond B|401:N07(LIG) B|401:C08(LIG)
    add a default BCOR for bond B|401:C08(LIG) B|401:C09(LIG)
    add a default BCOR for bond B|401:C09(LIG) B|401:O10(LIG)

 Have added a total of        22 BCORrels for bonds which do not already have a BCORrel


 No bonds (for potential BCORrel addition) were skipped due to both atoms having constant/fixed B(iso)


 ----------- Deactivate hydrogen-free planes if hydrogen atoms present

 A total of       0 planes have been deactivated because they are duplicated hydrogen-free and with-hydrogen forms.

 ----------- Handle peptide omega angle(s)

      Torsion angles split into peptide omega and other
         Number of peptide omega angles is       698
         Number of other torsion is             1269
      Peptide omega angles are monitored rather than directly restrained (PLANE used instead).

 ----------- Fix cis-PRO

 Now apply the fix to get correct restraints for cis PRO residues
   Have got      11 bonds records and      26 angles
   from GEOMETRY cards for the special PROC residue
   Weight for ideal-distance contact term=     4.000

 ----------- Prepare SSBOND/LINK for output PDB file


 ----------- Prepare for QM


 ----------- Prepare symmetry in utility distance restraints


 ############### Setting up Rhofit contacts (if needed)

 No ligand-(fixed protein) contact term will be used

 ############### Working out initial function and gradient


   MaxLik Scaling Cycle 0002
 
 Initial functional value = 4.155474E+05 rms gradient=      33.9664
 X-ray contribution to function value = 3.718116E+05
 Geometry contribution (inc ncs)      =      43735.8
 Geometry function summary
     term                           number     weight       rms        GooF      function     fn/numb
     ================================================================================================
     BOND bond lengths (angs)       5652       2.000      0.0081       0.406      1863.71       0.330
     ANGL bond angles (degs)        7669       2.000        1.02       0.610      5709.96       0.745
     TORS split-harmonic tors       1269       0.000       18.44       1.160         0.00       0.000
     SINTOR sinusoidal tors         1269       2.000       18.44                  2545.41       2.006
     PLAN general plane (angs)       963       5.000      0.0151       0.753      2727.77       2.833
     BCORrelations (angs^2)         5652      10.000       1.081       0.386      8412.85       1.488
     CONT (bad contacts)               0       5.000                                 0.00
     IDEAL(ideal-dist contact)      5513       4.000                             12036.39
     CHIRAL (gelly semiharmon)       740       5.000                                63.68
     SIM similarity restraint for NCS and/or TARGET                              10376.00
     OCCSUM restraint on sum of occupanies                                           0.00       as none defined
     DISTAN utility restraints interatomic distance                                  0.00       as none defined
     UTILANGLE utility angle restraints                                              0.00       as none defined
     UTILTOR utility restraints on torsion angles                                    0.00       as none defined
     Peptide omega torsion angles                           3.04 degs - monitor only

 Weighted rms      0.0081         1.024  (temporary rms bond and angle for BUSTER to pick up!)
 Weighted intra-residual rms(bond) for StdProtein (excluding H)      0.0080
 Weighted intra-residual rms(bond) for Buffer     (excluding H)      0.0031

 ############### Running initial check


 Initial Geometry Sanity Check

 Check for duplicate bonds: no duplicates found
 
 Check for duplicate or missing bond angles: all are OK
 Check for bond between an atom and itself not involving symmetry:  all are OK
   There are no bad contacts where Delta/sigma is greater than     6.5
   Worst contact dist=   3.006 std=   3.836 sigma=   0.217 abs(Delta/sigma)=    3.826 ats A|1:CB (SER) B|1:CB (SER)
   It is likely that the geometry libraries, sequence info and links match the input molecule
 GEOMETRY CHECK OVERALL:  PASS


 ############### Creating geometry/screen report


 ############### Option "tao" - run Tao optimizer

 TAO: Toolkit for Advanced Optimization. Reference: 
     Munson, T., Sarich, J., Wild, S., Benson, S., McInnes, L.C. (2018)
     "TAO User Manual (Revision 3.9)", Mathematics and Computer Science
     Division, Argonne National Laboratory
     ANL/MCS-TM-322, http://www.mcs.anl.gov/petsc/
 
 Have found -keeppdb flag so will write out a .pdb file every 1000000 its during opt
 Will apply TAO optimization 
 Maximum number of iterations      100
 Convergence test on Grms       4.0000 (-glim)
 Limit on rms displacement to original position (rmsD2init): NONE test inactive (-dlim)

    analysing argument no 0 : -autoncs with j=0
    analysing argument no 1 : -hydrogens_input_occ2zero with j=0
    analysing argument no 2 : -hydrogens_output_occ2heavy with j=0
    analysing argument no 3 : -screen with j=0
    analysing argument no 4 : 100 with j=0
    analysing argument no 5 : -screen_sigma with j=0
    analysing argument no 6 : 3.0 with j=0
    analysing argument no 7 : -keeppdb with j=0
    analysing argument no 8 : 1000000 with j=0
    analysing argument no 9 : -taomonitor_verbose with j=0
    found argument starting with "-tao" : -taomonitor_verbose
    analysing argument no 10 : 2 with j=1
    found value after -tao/-options_/-viewer_ argument : "2"
 PETSC/TAO: setting option "-taomonitor_verbose" = "2"


   MaxLik Scaling Cycle 0003
   Iters     Tot_Funct    Xray_Funct    Geom_Funct          Grms     rmsD2init     maxD2init             R         Rfree           LLG       LLGfree     rms(bond)    rms(angle)       Opttag_H
   Iters             K   B_IMPF_FRAG        K_MISS   B_TEMP_MISS   K_IMPF_MISS   B_IMPF_MISS        K_SOLV   B_TEMP_SOLV   K_IMPF_SOLV   B_IMPF_SOLV        B_TEMP     B_TEMP_11     B_TEMP_12     B_TEMP_22     B_TEMP_13     B_TEMP_23     B_TEMP_33      K_WILSON      B_WILSON      FGCYCLES       Scatag_H
   Iters          BOND         ANGLE      IMPROPER       TORSION    SINTORSION      TRIGONAL        PLANAR  BCORRELATION       CONTACT     IDEALDIST        CHIRAL    SIMILARITY       OCCSSUM  UTILDISTANCE     UTILANGLE   UTILTORSION        RHOFIT        ADP-01        ADP-02        ADP-03        ADP-04        ADP-05        ADP-06        ADP-07        ADP-08        ADP-09        ADP-10      RHOFITPC            QM       Funtag_H
       0  4.155474E+05  3.718116E+05       43735.8       33.9664  0.000000E+00  0.000000E+00      0.189082      0.234153  0.000000E+00  0.000000E+00    0.00814778       1.02363       Opttag_D
       0       78.5323       1.42812       1.00000  0.000000E+00       1.00000  0.000000E+00      0.882781       83.1775       1.00000       32.5208  0.000000E+00    -0.0551980  0.000000E+00    -0.0551980  0.000000E+00  0.000000E+00      0.110396       79.6522      0.868813        2       Scatag_D
       0       1863.71       5709.96  0.000000E+00  0.000000E+00       2545.41  0.000000E+00       2727.77       8412.85  0.000000E+00       12036.4       63.6843       10376.0 -1.000000E+10 -1.000000E+10 -1.000000E+10 -1.000000E+10 -1.000000E+10  0.000000E+00  0.000000E+00  0.000000E+00  0.000000E+00  0.000000E+00  0.000000E+00  0.000000E+00  0.000000E+00  0.000000E+00  0.000000E+00  0.000000E+00  0.000000E+00       Funtag_D
   Geometry Restraint Deviations SCREEN Information

 

 An atom has moved more than     1.50 A from the initial/last
 position - if this was an accepted trial point, probably need to update the
 non-bonded contact list.


   MaxLik Scaling Cycle 0004

   MaxLik Scaling Cycle 0005

   MaxLik Scaling Cycle 0006

   Wilson plot

   Statistics in resolution bins after refinement cycle 0001
```


|  |  |
| --- | --- |
|  | - Rxpct factors plot- Rxpct factors table- <Log-Lik Gain>- Ln Sigma A- Correlation Coefficients |


```
   Iters     Tot_Funct    Xray_Funct    Geom_Funct          Grms     rmsD2init     maxD2init             R         Rfree           LLG       LLGfree     rms(bond)    rms(angle)
   Iters             K   B_IMPF_FRAG        K_MISS   B_TEMP_MISS   K_IMPF_MISS   B_IMPF_MISS        K_SOLV   B_TEMP_SOLV   K_IMPF_SOLV   B_IMPF_SOLV        B_TEMP     B_TEMP_11     B_TEMP_12     B_TEMP_22     B_TEMP_13     B_TEMP_23     B_TEMP_33      K_WILSON      B_WILSON      FGCYCLES
   Iters          BOND         ANGLE      IMPROPER       TORSION    SINTORSION      TRIGONAL        PLANAR  BCORRELATION       CONTACT     IDEALDIST        CHIRAL    SIMILARITY       OCCSSUM  UTILDISTANCE     UTILANGLE   UTILTORSION        RHOFIT        ADP-01        ADP-02        ADP-03        ADP-04        ADP-05        ADP-06        ADP-07        ADP-08        ADP-09        ADP-10      RHOFITPC            QM
       1  4.153172E+05  3.717758E+05       43541.4       27.0755  2.926759E-04    0.00224969      0.189081      0.234134  8.600840E-05  1.475757E-04    0.00815165       1.02356       Opttag_D
       1       78.5157       1.42554       1.00000  0.000000E+00       1.00000  0.000000E+00      0.882813       83.0860       1.00000       32.5232  0.000000E+00    -0.0492558  0.000000E+00    -0.0492558  0.000000E+00  0.000000E+00     0.0985117       79.6522      0.868813        3       Scatag_D
       1       1865.48       5709.08  0.000000E+00  0.000000E+00       2545.41  0.000000E+00       2728.06       8220.17  0.000000E+00       12036.3       63.3171       10373.5 -1.000000E+10 -1.000000E+10 -1.000000E+10 -1.000000E+10 -1.000000E+10  0.000000E+00  0.000000E+00  0.000000E+00  0.000000E+00  0.000000E+00  0.000000E+00  0.000000E+00  0.000000E+00  0.000000E+00  0.000000E+00  0.000000E+00  0.000000E+00       Funtag_D
   Geometry Restraint Deviations SCREEN Information

 

   MaxLik Scaling Cycle 0007

   Wilson plot

   Statistics in resolution bins after refinement cycle 0002
```


|  |  |
| --- | --- |
|  | - Rxpct factors plot- Rxpct factors table- <Log-Lik Gain>- Ln Sigma A- Correlation Coefficients |


```
   Iters     Tot_Funct    Xray_Funct    Geom_Funct          Grms     rmsD2init     maxD2init             R         Rfree           LLG       LLGfree     rms(bond)    rms(angle)
   Iters             K   B_IMPF_FRAG        K_MISS   B_TEMP_MISS   K_IMPF_MISS   B_IMPF_MISS        K_SOLV   B_TEMP_SOLV   K_IMPF_SOLV   B_IMPF_SOLV        B_TEMP     B_TEMP_11     B_TEMP_12     B_TEMP_22     B_TEMP_13     B_TEMP_23     B_TEMP_33      K_WILSON      B_WILSON      FGCYCLES
   Iters          BOND         ANGLE      IMPROPER       TORSION    SINTORSION      TRIGONAL        PLANAR  BCORRELATION       CONTACT     IDEALDIST        CHIRAL    SIMILARITY       OCCSSUM  UTILDISTANCE     UTILANGLE   UTILTORSION        RHOFIT        ADP-01        ADP-02        ADP-03        ADP-04        ADP-05        ADP-06        ADP-07        ADP-08        ADP-09        ADP-10      RHOFITPC            QM
       2  4.151636E+05  3.717445E+05       43419.1       23.7539  6.498794E-04    0.00416626      0.189088      0.234149  1.612834E-04  1.716343E-04    0.00816107       1.02398       Opttag_D
       2       78.4896       1.42235       1.00000  0.000000E+00       1.00000  0.000000E+00      0.882818       82.9793       1.00000       32.5214  0.000000E+00    -0.0412819  0.000000E+00    -0.0412819  0.000000E+00  0.000000E+00     0.0825638       79.6522      0.868813        1       Scatag_D
       2       1869.80       5713.78  0.000000E+00  0.000000E+00       2545.60  0.000000E+00       2729.35       8089.66  0.000000E+00       12036.1       63.0479       10371.8 -1.000000E+10 -1.000000E+10 -1.000000E+10 -1.000000E+10 -1.000000E+10  0.000000E+00  0.000000E+00  0.000000E+00  0.000000E+00  0.000000E+00  0.000000E+00  0.000000E+00  0.000000E+00  0.000000E+00  0.000000E+00  0.000000E+00  0.000000E+00       Funtag_D
   Geometry Restraint Deviations SCREEN Information

 
       3  4.150601E+05  3.717184E+05       43341.7       21.0966    0.00100219    0.00702784      0.189100      0.234170  2.236360E-04  1.355051E-04    0.00817554       1.02411       Opttag_D
       3       78.4634       1.41943       1.00000  0.000000E+00       1.00000  0.000000E+00      0.882812       82.8803       1.00000       32.5200  0.000000E+00    -0.0341519  0.000000E+00    -0.0341519  0.000000E+00  0.000000E+00     0.0683039       79.6522      0.868813        1       Scatag_D
       3       1876.44       5715.28  0.000000E+00  0.000000E+00       2545.72  0.000000E+00       2731.45       8001.75  0.000000E+00       12036.1       62.8414       10372.1 -1.000000E+10 -1.000000E+10 -1.000000E+10 -1.000000E+10 -1.000000E+10  0.000000E+00  0.000000E+00  0.000000E+00  0.000000E+00  0.000000E+00  0.000000E+00  0.000000E+00  0.000000E+00  0.000000E+00  0.000000E+00  0.000000E+00  0.000000E+00       Funtag_D
       4  4.149740E+05  3.716885E+05       43285.5       23.4143    0.00150174     0.0112623      0.189122      0.234200  2.953802E-04  6.531361E-05    0.00819472       1.02503       Opttag_D
       4       78.4251       1.41545       1.00000  0.000000E+00       1.00000  0.000000E+00      0.882797       82.7429       1.00000       32.5188  0.000000E+00    -0.0242003  0.000000E+00    -0.0242003  0.000000E+00  0.000000E+00     0.0484005       79.6522      0.868813        1       Scatag_D
       4       1885.25       5725.49  0.000000E+00  0.000000E+00       2545.86  0.000000E+00       2735.33       7921.34  0.000000E+00       12036.2       62.6543       10373.3 -1.000000E+10 -1.000000E+10 -1.000000E+10 -1.000000E+10 -1.000000E+10  0.000000E+00  0.000000E+00  0.000000E+00  0.000000E+00  0.000000E+00  0.000000E+00  0.000000E+00  0.000000E+00  0.000000E+00  0.000000E+00  0.000000E+00  0.000000E+00       Funtag_D
       5  4.149019E+05  3.716710E+05       43230.9       20.9963    0.00190679     0.0149490      0.189147      0.234221  3.372546E-04 -1.537763E-05    0.00820360       1.02504       Opttag_D
       5       78.3923       1.41233       1.00000  0.000000E+00       1.00000  0.000000E+00      0.882775       82.6331       1.00000       32.5184  0.000000E+00    -0.0159591  0.000000E+00    -0.0159591  0.000000E+00  0.000000E+00     0.0319182       79.6522      0.868813        1       Scatag_D
       5       1889.33       5725.70  0.000000E+00  0.000000E+00       2545.94  0.000000E+00       2739.43       7856.50  0.000000E+00       12036.2       62.6198       10375.1 -1.000000E+10 -1.000000E+10 -1.000000E+10 -1.000000E+10 -1.000000E+10  0.000000E+00  0.000000E+00  0.000000E+00  0.000000E+00  0.000000E+00  0.000000E+00  0.000000E+00  0.000000E+00  0.000000E+00  0.000000E+00  0.000000E+00  0.000000E+00       Funtag_D
       6  4.148322E+05  3.716614E+05       43170.8       18.4031    0.00223792     0.0181186      0.189167      0.234236  3.603455E-04 -8.457699E-05    0.00820555       1.02504       Opttag_D
       6       78.3640       1.40984       1.00000  0.000000E+00       1.00000  0.000000E+00      0.882750       82.5447       1.00000       32.5182  0.000000E+00   -0.00928378  0.000000E+00   -0.00928378  0.000000E+00  0.000000E+00     0.0185676       79.6522      0.868813        1       Scatag_D
       6       1890.24       5725.64  0.000000E+00  0.000000E+00       2546.02  0.000000E+00       2743.39       7790.79  0.000000E+00       12035.5       62.6948       10376.5 -1.000000E+10 -1.000000E+10 -1.000000E+10 -1.000000E+10 -1.000000E+10  0.000000E+00  0.000000E+00  0.000000E+00  0.000000E+00  0.000000E+00  0.000000E+00  0.000000E+00  0.000000E+00  0.000000E+00  0.000000E+00  0.000000E+00  0.000000E+00       Funtag_D
       7  4.147435E+05  3.716561E+05       43087.4       22.2350    0.00294404     0.0245505      0.189218      0.234276  3.730561E-04 -2.553018E-04    0.00821084       1.02619       Opttag_D
       7       78.3059       1.40511       1.00000  0.000000E+00       1.00000  0.000000E+00      0.882693       82.3665       1.00000       32.5200  0.000000E+00    0.00413852  0.000000E+00    0.00413852  0.000000E+00  0.000000E+00   -0.00827704       79.6522      0.868813        1       Scatag_D
       7       1892.67       5738.54  0.000000E+00  0.000000E+00       2546.15  0.000000E+00       2752.20       7682.39  0.000000E+00       12033.9       62.8923       10378.6 -1.000000E+10 -1.000000E+10 -1.000000E+10 -1.000000E+10 -1.000000E+10  0.000000E+00  0.000000E+00  0.000000E+00  0.000000E+00  0.000000E+00  0.000000E+00  0.000000E+00  0.000000E+00  0.000000E+00  0.000000E+00  0.000000E+00  0.000000E+00       Funtag_D
       8  4.146904E+05  3.716664E+05       43024.0       25.5009    0.00354155     0.0296220      0.189268      0.234323  3.484117E-04 -4.022988E-04    0.00821443       1.02748       Opttag_D
       8       78.2589       1.40155       1.00000  0.000000E+00       1.00000  0.000000E+00      0.882644       82.2210       1.00000       32.5229  0.000000E+00     0.0152459  0.000000E+00     0.0152459  0.000000E+00  0.000000E+00    -0.0304917       79.6522      0.868813        1       Scatag_D
       8       1894.33       5752.98  0.000000E+00  0.000000E+00       2546.29  0.000000E+00       2759.10       7596.06  0.000000E+00       12032.7       62.9868       10379.5 -1.000000E+10 -1.000000E+10 -1.000000E+10 -1.000000E+10 -1.000000E+10  0.000000E+00  0.000000E+00  0.000000E+00  0.000000E+00  0.000000E+00  0.000000E+00  0.000000E+00  0.000000E+00  0.000000E+00  0.000000E+00  0.000000E+00  0.000000E+00       Funtag_D
       9  4.146393E+05  3.716677E+05       42971.6       15.5108    0.00362851     0.0302833      0.189276      0.234335  3.453046E-04 -4.323926E-04    0.00821246       1.02600       Opttag_D
       9       78.2510       1.40090       1.00000  0.000000E+00       1.00000  0.000000E+00      0.882634       82.1963       1.00000       32.5246  0.000000E+00     0.0169526  0.000000E+00     0.0169526  0.000000E+00  0.000000E+00    -0.0339052       79.6522      0.868813        1       Scatag_D
       9       1893.42       5736.42  0.000000E+00  0.000000E+00       2546.30  0.000000E+00       2759.73       7562.74  0.000000E+00       12031.7       62.9636       10378.4 -1.000000E+10 -1.000000E+10 -1.000000E+10 -1.000000E+10 -1.000000E+10  0.000000E+00  0.000000E+00  0.000000E+00  0.000000E+00  0.000000E+00  0.000000E+00  0.000000E+00  0.000000E+00  0.000000E+00  0.000000E+00  0.000000E+00  0.000000E+00       Funtag_D
      10  4.145882E+05  3.716726E+05       42915.5       13.0676    0.00394151     0.0326908      0.189299      0.234364  3.334414E-04 -5.136626E-04    0.00821599       1.02598       Opttag_D
      10       78.2267       1.39892       1.00000  0.000000E+00       1.00000  0.000000E+00      0.882607       82.1171       1.00000       32.5293  0.000000E+00     0.0224729  0.000000E+00     0.0224729  0.000000E+00  0.000000E+00    -0.0449458       79.6522      0.868813        1       Scatag_D
      10       1895.05       5736.20  0.000000E+00  0.000000E+00       2546.40  0.000000E+00       2761.93       7506.48  0.000000E+00       12029.9       62.9161       10376.7 -1.000000E+10 -1.000000E+10 -1.000000E+10 -1.000000E+10 -1.000000E+10  0.000000E+00  0.000000E+00  0.000000E+00  0.000000E+00  0.000000E+00  0.000000E+00  0.000000E+00  0.000000E+00  0.000000E+00  0.000000E+00  0.000000E+00  0.000000E+00       Funtag_D
      11  4.145475E+05  3.716824E+05       42865.2       14.7429    0.00440296     0.0362309      0.189337      0.234394  3.101387E-04 -6.079127E-04    0.00822320       1.02644       Opttag_D
      11       78.1941       1.39635       1.00000  0.000000E+00       1.00000  0.000000E+00      0.882571       82.0106       1.00000       32.5355  0.000000E+00     0.0298153  0.000000E+00     0.0298153  0.000000E+00  0.000000E+00    -0.0596305       79.6522      0.868813        1       Scatag_D
      11       1898.37       5741.33  0.000000E+00  0.000000E+00       2546.55  0.000000E+00       2764.71       7447.82  0.000000E+00       12027.8       62.8997       10375.7 -1.000000E+10 -1.000000E+10 -1.000000E+10 -1.000000E+10 -1.000000E+10  0.000000E+00  0.000000E+00  0.000000E+00  0.000000E+00  0.000000E+00  0.000000E+00  0.000000E+00  0.000000E+00  0.000000E+00  0.000000E+00  0.000000E+00  0.000000E+00       Funtag_D
      12  4.145119E+05  3.717105E+05       42801.4       20.5154    0.00533212     0.0429392      0.189413      0.234441  2.427725E-04 -7.576379E-04    0.00823476       1.02792       Opttag_D
      12       78.1351       1.39185       1.00000  0.000000E+00       1.00000  0.000000E+00      0.882500       81.8144       1.00000       32.5487  0.000000E+00     0.0429376  0.000000E+00     0.0429376  0.000000E+00  0.000000E+00    -0.0858752       79.6522      0.868813        1       Scatag_D
      12       1903.72       5757.88  0.000000E+00  0.000000E+00       2547.03  0.000000E+00       2768.79       7362.72  0.000000E+00       12023.7       62.8953       10374.7 -1.000000E+10 -1.000000E+10 -1.000000E+10 -1.000000E+10 -1.000000E+10  0.000000E+00  0.000000E+00  0.000000E+00  0.000000E+00  0.000000E+00  0.000000E+00  0.000000E+00  0.000000E+00  0.000000E+00  0.000000E+00  0.000000E+00  0.000000E+00       Funtag_D
      13  4.144727E+05  3.717139E+05       42758.9       10.4512    0.00550596     0.0441511      0.189423      0.234441  2.345106E-04 -7.606969E-04    0.00822995       1.02692       Opttag_D
      13       78.1267       1.39114       1.00000  0.000000E+00       1.00000  0.000000E+00      0.882485       81.7843       1.00000       32.5509  0.000000E+00     0.0445100  0.000000E+00     0.0445100  0.000000E+00  0.000000E+00    -0.0890200       79.6522      0.868813        1       Scatag_D
      13       1901.49       5746.62  0.000000E+00  0.000000E+00       2547.18  0.000000E+00       2768.24       7335.44  0.000000E+00       12023.1       62.9251       10373.9 -1.000000E+10 -1.000000E+10 -1.000000E+10 -1.000000E+10 -1.000000E+10  0.000000E+00  0.000000E+00  0.000000E+00  0.000000E+00  0.000000E+00  0.000000E+00  0.000000E+00  0.000000E+00  0.000000E+00  0.000000E+00  0.000000E+00  0.000000E+00       Funtag_D
      14  4.144483E+05  3.717144E+05       42733.9       10.0651    0.00567704     0.0452549      0.189430      0.234435  2.333101E-04 -7.430870E-04    0.00822706       1.02703       Opttag_D
      14       78.1205       1.39052       1.00000  0.000000E+00       1.00000  0.000000E+00      0.882471       81.7594       1.00000       32.5529  0.000000E+00     0.0454111  0.000000E+00     0.0454111  0.000000E+00  0.000000E+00    -0.0908221       79.6522      0.868813        1       Scatag_D
      14       1900.16       5747.93  0.000000E+00  0.000000E+00       2547.42  0.000000E+00       2766.81       7313.39  0.000000E+00       12022.1       62.9740       10373.0 -1.000000E+10 -1.000000E+10 -1.000000E+10 -1.000000E+10 -1.000000E+10  0.000000E+00  0.000000E+00  0.000000E+00  0.000000E+00  0.000000E+00  0.000000E+00  0.000000E+00  0.000000E+00  0.000000E+00  0.000000E+00  0.000000E+00  0.000000E+00       Funtag_D
      15  4.144219E+05  3.717192E+05       42702.7       11.1076    0.00606776     0.0476944      0.189451      0.234433  2.218000E-04 -7.464767E-04    0.00822604       1.02723       Opttag_D
      15       78.1062       1.38917       1.00000  0.000000E+00       1.00000  0.000000E+00      0.882441       81.7008       1.00000       32.5580  0.000000E+00     0.0479647  0.000000E+00     0.0479647  0.000000E+00  0.000000E+00    -0.0959295       79.6522      0.868813        1       Scatag_D
      15       1899.68       5750.13  0.000000E+00  0.000000E+00       2547.89  0.000000E+00       2765.21       7283.94  0.000000E+00       12020.6       63.0620       10372.2 -1.000000E+10 -1.000000E+10 -1.000000E+10 -1.000000E+10 -1.000000E+10  0.000000E+00  0.000000E+00  0.000000E+00  0.000000E+00  0.000000E+00  0.000000E+00  0.000000E+00  0.000000E+00  0.000000E+00  0.000000E+00  0.000000E+00  0.000000E+00       Funtag_D
      16  4.144071E+05  3.717411E+05       42666.0       24.6631    0.00709861     0.0547866      0.189502      0.234446  1.693334E-04 -8.228688E-04    0.00823510       1.02961       Opttag_D
      16       78.0726       1.38612       1.00000  0.000000E+00       1.00000  0.000000E+00      0.882370       81.5546       1.00000       32.5715  0.000000E+00     0.0544322  0.000000E+00     0.0544322  0.000000E+00  0.000000E+00     -0.108864       79.6522      0.868813        1       Scatag_D
      16       1903.87       5776.83  0.000000E+00  0.000000E+00       2549.21  0.000000E+00       2762.63       7221.51  0.000000E+00       12017.5       63.2182       10371.2 -1.000000E+10 -1.000000E+10 -1.000000E+10 -1.000000E+10 -1.000000E+10  0.000000E+00  0.000000E+00  0.000000E+00  0.000000E+00  0.000000E+00  0.000000E+00  0.000000E+00  0.000000E+00  0.000000E+00  0.000000E+00  0.000000E+00  0.000000E+00       Funtag_D
      17  4.143710E+05  3.717406E+05       42630.3       9.96507    0.00721654     0.0560579      0.189503      0.234448  1.704633E-04 -8.458526E-04    0.00823111       1.02758       Opttag_D
      17       78.0710       1.38583       1.00000  0.000000E+00       1.00000  0.000000E+00      0.882365       81.5402       1.00000       32.5729  0.000000E+00     0.0547687  0.000000E+00     0.0547687  0.000000E+00  0.000000E+00     -0.109537       79.6522      0.868813        1       Scatag_D
      17       1902.03       5754.02  0.000000E+00  0.000000E+00       2549.32  0.000000E+00       2762.53       7210.82  0.000000E+00       12017.4       63.2234       10371.0 -1.000000E+10 -1.000000E+10 -1.000000E+10 -1.000000E+10 -1.000000E+10  0.000000E+00  0.000000E+00  0.000000E+00  0.000000E+00  0.000000E+00  0.000000E+00  0.000000E+00  0.000000E+00  0.000000E+00  0.000000E+00  0.000000E+00  0.000000E+00       Funtag_D
      18  4.143534E+05  3.717417E+05       42611.7       7.49155    0.00745543     0.0584769      0.189511      0.234455  1.678505E-04 -8.921509E-04    0.00823201       1.02758       Opttag_D
      18       78.0668       1.38523       1.00000  0.000000E+00       1.00000  0.000000E+00      0.882352       81.5097       1.00000       32.5758  0.000000E+00     0.0556827  0.000000E+00     0.0556827  0.000000E+00  0.000000E+00     -0.111365       79.6522      0.868813        1       Scatag_D
      18       1902.44       5754.08  0.000000E+00  0.000000E+00       2549.61  0.000000E+00       2762.34       7192.35  0.000000E+00       12017.0       63.2287       10370.6 -1.000000E+10 -1.000000E+10 -1.000000E+10 -1.000000E+10 -1.000000E+10  0.000000E+00  0.000000E+00  0.000000E+00  0.000000E+00  0.000000E+00  0.000000E+00  0.000000E+00  0.000000E+00  0.000000E+00  0.000000E+00  0.000000E+00  0.000000E+00       Funtag_D
      19  4.143349E+05  3.717475E+05       42587.4       9.55781    0.00800115     0.0638699      0.189528      0.234469  1.539395E-04 -9.811097E-04    0.00823707       1.02788       Opttag_D
      19       78.0571       1.38397       1.00000  0.000000E+00       1.00000  0.000000E+00      0.882323       81.4436       1.00000       32.5823  0.000000E+00     0.0576628  0.000000E+00     0.0576628  0.000000E+00  0.000000E+00     -0.115326       79.6522      0.868813        1       Scatag_D
      19       1904.78       5757.41  0.000000E+00  0.000000E+00       2550.28  0.000000E+00       2761.62       7163.88  0.000000E+00       12015.9       63.2491       10370.3 -1.000000E+10 -1.000000E+10 -1.000000E+10 -1.000000E+10 -1.000000E+10  0.000000E+00  0.000000E+00  0.000000E+00  0.000000E+00  0.000000E+00  0.000000E+00  0.000000E+00  0.000000E+00  0.000000E+00  0.000000E+00  0.000000E+00  0.000000E+00       Funtag_D
      20  4.143169E+05  3.717536E+05       42563.3       16.6578    0.00900824     0.0737678      0.189553      0.234492  1.392517E-04   -0.00112604    0.00824047       1.02930       Opttag_D
      20       78.0461       1.38205       1.00000  0.000000E+00       1.00000  0.000000E+00      0.882273       81.3376       1.00000       32.5935  0.000000E+00     0.0597576  0.000000E+00     0.0597576  0.000000E+00  0.000000E+00     -0.119515       79.6522      0.868813        1       Scatag_D
      20       1906.36       5773.33  0.000000E+00  0.000000E+00       2551.61  0.000000E+00       2760.09       7124.92  0.000000E+00       12013.7       63.2389       10370.0 -1.000000E+10 -1.000000E+10 -1.000000E+10 -1.000000E+10 -1.000000E+10  0.000000E+00  0.000000E+00  0.000000E+00  0.000000E+00  0.000000E+00  0.000000E+00  0.000000E+00  0.000000E+00  0.000000E+00  0.000000E+00  0.000000E+00  0.000000E+00       Funtag_D
      21  4.142940E+05  3.717566E+05       42537.4       9.05992    0.00951345     0.0787585      0.189566      0.234502  1.321196E-04   -0.00119764    0.00824052       1.02852       Opttag_D
      21       78.0424       1.38120       1.00000  0.000000E+00       1.00000  0.000000E+00      0.882248       81.2891       1.00000       32.5994  0.000000E+00     0.0604069  0.000000E+00     0.0604069  0.000000E+00  0.000000E+00     -0.120814       79.6522      0.868813        1       Scatag_D
      21       1906.38       5764.55  0.000000E+00  0.000000E+00       2552.28  0.000000E+00       2759.23       7109.23  0.000000E+00       12012.5       63.2225       10370.0 -1.000000E+10 -1.000000E+10 -1.000000E+10 -1.000000E+10 -1.000000E+10  0.000000E+00  0.000000E+00  0.000000E+00  0.000000E+00  0.000000E+00  0.000000E+00  0.000000E+00  0.000000E+00  0.000000E+00  0.000000E+00  0.000000E+00  0.000000E+00       Funtag_D
      22  4.142766E+05  3.717529E+05       42523.7       6.82476    0.00999222     0.0843839      0.189569      0.234507  1.410877E-04   -0.00125319    0.00824065       1.02855       Opttag_D
      22       78.0432       1.38055       1.00000  0.000000E+00       1.00000  0.000000E+00      0.882225       81.2528       1.00000       32.6041  0.000000E+00     0.0600927  0.000000E+00     0.0600927  0.000000E+00  0.000000E+00     -0.120185       79.6522      0.868813        1       Scatag_D
      22       1906.44       5764.93  0.000000E+00  0.000000E+00       2552.93  0.000000E+00       2758.21       7096.67  0.000000E+00       12011.0       63.1739       10370.3 -1.000000E+10 -1.000000E+10 -1.000000E+10 -1.000000E+10 -1.000000E+10  0.000000E+00  0.000000E+00  0.000000E+00  0.000000E+00  0.000000E+00  0.000000E+00  0.000000E+00  0.000000E+00  0.000000E+00  0.000000E+00  0.000000E+00  0.000000E+00       Funtag_D
      23  4.142598E+05  3.717432E+05       42516.6       9.02905     0.0106361     0.0921319      0.189569      0.234513  1.641786E-04   -0.00132628    0.00823969       1.02903       Opttag_D
      23       78.0475       1.37981       1.00000  0.000000E+00       1.00000  0.000000E+00      0.882197       81.2115       1.00000       32.6101  0.000000E+00     0.0591471  0.000000E+00     0.0591471  0.000000E+00  0.000000E+00     -0.118294       79.6522      0.868813        1       Scatag_D
      23       1905.99       5770.35  0.000000E+00  0.000000E+00       2553.84  0.000000E+00       2757.07       7086.13  0.000000E+00       12009.4       63.1155       10370.8 -1.000000E+10 -1.000000E+10 -1.000000E+10 -1.000000E+10 -1.000000E+10  0.000000E+00  0.000000E+00  0.000000E+00  0.000000E+00  0.000000E+00  0.000000E+00  0.000000E+00  0.000000E+00  0.000000E+00  0.000000E+00  0.000000E+00  0.000000E+00       Funtag_D
      24  4.142422E+05  3.717225E+05       42519.7       13.5095     0.0118688      0.107054      0.189567      0.234524  2.139618E-04   -0.00147973    0.00824575       1.02984       Opttag_D
      24       78.0601       1.37867       1.00000  0.000000E+00       1.00000  0.000000E+00      0.882146       81.1439       1.00000       32.6213  0.000000E+00     0.0569662  0.000000E+00     0.0569662  0.000000E+00  0.000000E+00     -0.113932       79.6522      0.868813        1       Scatag_D
      24       1908.80       5779.42  0.000000E+00  0.000000E+00       2555.63  0.000000E+00       2755.70       7077.40  0.000000E+00       12007.1       63.0278       10372.6 -1.000000E+10 -1.000000E+10 -1.000000E+10 -1.000000E+10 -1.000000E+10  0.000000E+00  0.000000E+00  0.000000E+00  0.000000E+00  0.000000E+00  0.000000E+00  0.000000E+00  0.000000E+00  0.000000E+00  0.000000E+00  0.000000E+00  0.000000E+00       Funtag_D
      25  4.142224E+05  3.717087E+05       42513.7       7.97972     0.0123407      0.113179      0.189559      0.234527  2.470799E-04   -0.00153768    0.00824200       1.02963       Opttag_D
      25       78.0697       1.37845       1.00000  0.000000E+00       1.00000  0.000000E+00      0.882128       81.1291       1.00000       32.6248  0.000000E+00     0.0555149  0.000000E+00     0.0555149  0.000000E+00  0.000000E+00     -0.111030       79.6522      0.868813        1       Scatag_D
      25       1907.07       5777.04  0.000000E+00  0.000000E+00       2556.25  0.000000E+00       2755.49       7075.23  0.000000E+00       12006.9       63.0006       10372.8 -1.000000E+10 -1.000000E+10 -1.000000E+10 -1.000000E+10 -1.000000E+10  0.000000E+00  0.000000E+00  0.000000E+00  0.000000E+00  0.000000E+00  0.000000E+00  0.000000E+00  0.000000E+00  0.000000E+00  0.000000E+00  0.000000E+00  0.000000E+00       Funtag_D
      26  4.142060E+05  3.716912E+05       42514.8       7.13788     0.0127584      0.119028      0.189545      0.234526  2.890249E-04   -0.00159382    0.00824248       1.02979       Opttag_D
      26       78.0822       1.37843       1.00000  0.000000E+00       1.00000  0.000000E+00      0.882115       81.1253       1.00000       32.6269  0.000000E+00     0.0538214  0.000000E+00     0.0538214  0.000000E+00  0.000000E+00     -0.107643       79.6522      0.868813        1       Scatag_D
      26       1907.29       5778.85  0.000000E+00  0.000000E+00       2556.94  0.000000E+00       2755.66       7072.76  0.000000E+00       12007.3       62.9893       10373.0 -1.000000E+10 -1.000000E+10 -1.000000E+10 -1.000000E+10 -1.000000E+10  0.000000E+00  0.000000E+00  0.000000E+00  0.000000E+00  0.000000E+00  0.000000E+00  0.000000E+00  0.000000E+00  0.000000E+00  0.000000E+00  0.000000E+00  0.000000E+00       Funtag_D
      27  4.141890E+05  3.716689E+05       42520.2       8.61758     0.0134390      0.128468      0.189528      0.234527  3.424801E-04   -0.00168253    0.00824398       1.03008       Opttag_D
      27       78.1022       1.37854       1.00000  0.000000E+00       1.00000  0.000000E+00      0.882093       81.1205       1.00000       32.6303  0.000000E+00     0.0512310  0.000000E+00     0.0512310  0.000000E+00  0.000000E+00     -0.102462       79.6522      0.868813        1       Scatag_D
      27       1907.98       5782.06  0.000000E+00  0.000000E+00       2558.04  0.000000E+00       2756.06       7071.16  0.000000E+00       12008.2       62.9739       10373.7 -1.000000E+10 -1.000000E+10 -1.000000E+10 -1.000000E+10 -1.000000E+10  0.000000E+00  0.000000E+00  0.000000E+00  0.000000E+00  0.000000E+00  0.000000E+00  0.000000E+00  0.000000E+00  0.000000E+00  0.000000E+00  0.000000E+00  0.000000E+00       Funtag_D
      28  4.141741E+05  3.716352E+05       42538.8       13.4324     0.0147509      0.146595      0.189496      0.234525  4.231218E-04   -0.00182795    0.00825234       1.03039       Opttag_D
      28       78.1415       1.37910       1.00000  0.000000E+00       1.00000  0.000000E+00      0.882051       81.1179       1.00000       32.6370  0.000000E+00     0.0461520  0.000000E+00     0.0461520  0.000000E+00  0.000000E+00    -0.0923040       79.6522      0.868813        1       Scatag_D
      28       1911.85       5785.59  0.000000E+00  0.000000E+00       2560.16  0.000000E+00       2756.92       7075.14  0.000000E+00       12011.1       62.9067       10375.1 -1.000000E+10 -1.000000E+10 -1.000000E+10 -1.000000E+10 -1.000000E+10  0.000000E+00  0.000000E+00  0.000000E+00  0.000000E+00  0.000000E+00  0.000000E+00  0.000000E+00  0.000000E+00  0.000000E+00  0.000000E+00  0.000000E+00  0.000000E+00       Funtag_D
      29  4.141555E+05  3.716282E+05       42527.3       7.03999     0.0150947      0.151661      0.189485      0.234524  4.400692E-04   -0.00184507    0.00824808       1.03008       Opttag_D
      29       78.1536       1.37944       1.00000  0.000000E+00       1.00000  0.000000E+00      0.882041       81.1232       1.00000       32.6382  0.000000E+00     0.0445903  0.000000E+00     0.0445903  0.000000E+00  0.000000E+00    -0.0891806       79.6522      0.868813        1       Scatag_D
      29       1909.88       5782.15  0.000000E+00  0.000000E+00       2560.78  0.000000E+00       2757.09       7067.33  0.000000E+00       12012.1       62.8547       10375.1 -1.000000E+10 -1.000000E+10 -1.000000E+10 -1.000000E+10 -1.000000E+10  0.000000E+00  0.000000E+00  0.000000E+00  0.000000E+00  0.000000E+00  0.000000E+00  0.000000E+00  0.000000E+00  0.000000E+00  0.000000E+00  0.000000E+00  0.000000E+00       Funtag_D
      30  4.141413E+05  3.716210E+05       42520.3       6.51576     0.0155141      0.158044      0.189474      0.234522  4.572992E-04   -0.00184962    0.00824592       1.03009       Opttag_D
      30       78.1689       1.37996       1.00000  0.000000E+00       1.00000  0.000000E+00      0.882028       81.1328       1.00000       32.6394  0.000000E+00     0.0425682  0.000000E+00     0.0425682  0.000000E+00  0.000000E+00    -0.0851364       79.6522      0.868813        1       Scatag_D
      30       1908.88       5782.20  0.000000E+00  0.000000E+00       2561.52  0.000000E+00       2757.03       7059.22  0.000000E+00       12013.7       62.7806       10375.0 -1.000000E+10 -1.000000E+10 -1.000000E+10 -1.000000E+10 -1.000000E+10  0.000000E+00  0.000000E+00  0.000000E+00  0.000000E+00  0.000000E+00  0.000000E+00  0.000000E+00  0.000000E+00  0.000000E+00  0.000000E+00  0.000000E+00  0.000000E+00       Funtag_D
      31  4.141267E+05  3.716074E+05       42519.3       7.59234     0.0162358      0.168893      0.189454      0.234518  4.898524E-04   -0.00185573    0.00824555       1.03019       Opttag_D
      31       78.1947       1.38078       1.00000  0.000000E+00       1.00000  0.000000E+00      0.882009       81.1465       1.00000       32.6419  0.000000E+00     0.0391383  0.000000E+00     0.0391383  0.000000E+00  0.000000E+00    -0.0782766       79.6522      0.868813        1       Scatag_D
      31       1908.71       5783.36  0.000000E+00  0.000000E+00       2562.80  0.000000E+00       2756.80       7053.44  0.000000E+00       12016.4       62.7108       10375.1 -1.000000E+10 -1.000000E+10 -1.000000E+10 -1.000000E+10 -1.000000E+10  0.000000E+00  0.000000E+00  0.000000E+00  0.000000E+00  0.000000E+00  0.000000E+00  0.000000E+00  0.000000E+00  0.000000E+00  0.000000E+00  0.000000E+00  0.000000E+00       Funtag_D
      32  4.141142E+05  3.715758E+05       42538.4       15.9832     0.0178698      0.193679      0.189405      0.234500  5.656217E-04   -0.00184895    0.00825122       1.03111       Opttag_D
      32       78.2541       1.38268       1.00000  0.000000E+00       1.00000  0.000000E+00      0.881976       81.1768       1.00000       32.6479  0.000000E+00     0.0315134  0.000000E+00     0.0315134  0.000000E+00  0.000000E+00    -0.0630267       79.6522      0.868813        1       Scatag_D
      32       1911.34       5793.72  0.000000E+00  0.000000E+00       2565.71  0.000000E+00       2755.59       7050.68  0.000000E+00       12023.0       62.6129       10375.7 -1.000000E+10 -1.000000E+10 -1.000000E+10 -1.000000E+10 -1.000000E+10  0.000000E+00  0.000000E+00  0.000000E+00  0.000000E+00  0.000000E+00  0.000000E+00  0.000000E+00  0.000000E+00  0.000000E+00  0.000000E+00  0.000000E+00  0.000000E+00       Funtag_D
      33  4.140920E+05  3.715640E+05       42528.0       7.40865     0.0184539      0.202556      0.189388      0.234490  5.937969E-04   -0.00183746    0.00825040       1.03049       Opttag_D
      33       78.2754       1.38335       1.00000  0.000000E+00       1.00000  0.000000E+00      0.881967       81.1858       1.00000       32.6505  0.000000E+00     0.0288188  0.000000E+00     0.0288188  0.000000E+00  0.000000E+00    -0.0576375       79.6522      0.868813        1       Scatag_D
      33       1910.95       5786.71  0.000000E+00  0.000000E+00       2566.72  0.000000E+00       2755.09       7044.93  0.000000E+00       12025.4       62.6044       10375.6 -1.000000E+10 -1.000000E+10 -1.000000E+10 -1.000000E+10 -1.000000E+10  0.000000E+00  0.000000E+00  0.000000E+00  0.000000E+00  0.000000E+00  0.000000E+00  0.000000E+00  0.000000E+00  0.000000E+00  0.000000E+00  0.000000E+00  0.000000E+00       Funtag_D
      34  4.140797E+05  3.715519E+05       42527.9       5.79407     0.0188649      0.209252      0.189374      0.234478  6.229607E-04   -0.00181241    0.00825309       1.03051       Opttag_D
      34       78.2912       1.38378       1.00000  0.000000E+00       1.00000  0.000000E+00      0.881966       81.1908       1.00000       32.6520  0.000000E+00     0.0269302  0.000000E+00     0.0269302  0.000000E+00  0.000000E+00    -0.0538604       79.6522      0.868813        1       Scatag_D
      34       1912.20       5786.97  0.000000E+00  0.000000E+00       2567.50  0.000000E+00       2754.31       7041.98  0.000000E+00       12027.0       62.6204       10375.3 -1.000000E+10 -1.000000E+10 -1.000000E+10 -1.000000E+10 -1.000000E+10  0.000000E+00  0.000000E+00  0.000000E+00  0.000000E+00  0.000000E+00  0.000000E+00  0.000000E+00  0.000000E+00  0.000000E+00  0.000000E+00  0.000000E+00  0.000000E+00       Funtag_D
      35  4.140653E+05  3.715288E+05       42536.5       7.09330     0.0197222      0.223138      0.189342      0.234456  6.782518E-04   -0.00176239    0.00825795       1.03076       Opttag_D
      35       78.3238       1.38474       1.00000  0.000000E+00       1.00000  0.000000E+00      0.881964       81.2018       1.00000       32.6552  0.000000E+00     0.0231386  0.000000E+00     0.0231386  0.000000E+00  0.000000E+00    -0.0462772       79.6522      0.868813        1       Scatag_D
      35       1914.46       5789.76  0.000000E+00  0.000000E+00       2569.06  0.000000E+00       2753.00       7042.18  0.000000E+00       12030.5       62.6437       10374.9 -1.000000E+10 -1.000000E+10 -1.000000E+10 -1.000000E+10 -1.000000E+10  0.000000E+00  0.000000E+00  0.000000E+00  0.000000E+00  0.000000E+00  0.000000E+00  0.000000E+00  0.000000E+00  0.000000E+00  0.000000E+00  0.000000E+00  0.000000E+00       Funtag_D
      36  4.140514E+05  3.714917E+05       42559.7       12.9122     0.0212304      0.247835      0.189294      0.234411  7.672966E-04   -0.00165582    0.00826790       1.03140       Opttag_D
      36       78.3811       1.38655       1.00000  0.000000E+00       1.00000  0.000000E+00      0.881961       81.2217       1.00000       32.6610  0.000000E+00     0.0166894  0.000000E+00     0.0166894  0.000000E+00  0.000000E+00    -0.0333787       79.6522      0.868813        1       Scatag_D
      36       1919.07       5796.88  0.000000E+00  0.000000E+00       2571.69  0.000000E+00       2751.52       7046.90  0.000000E+00       12036.5       62.5285       10374.5 -1.000000E+10 -1.000000E+10 -1.000000E+10 -1.000000E+10 -1.000000E+10  0.000000E+00  0.000000E+00  0.000000E+00  0.000000E+00  0.000000E+00  0.000000E+00  0.000000E+00  0.000000E+00  0.000000E+00  0.000000E+00  0.000000E+00  0.000000E+00       Funtag_D
      37  4.140330E+05  3.714723E+05       42560.7       7.24336     0.0221182      0.262329      0.189265      0.234383  8.137609E-04   -0.00158811    0.00826669       1.03114       Opttag_D
      37       78.4144       1.38767       1.00000  0.000000E+00       1.00000  0.000000E+00      0.881961       81.2337       1.00000       32.6648  0.000000E+00     0.0131148  0.000000E+00     0.0131148  0.000000E+00  0.000000E+00    -0.0262296       79.6522      0.868813        1       Scatag_D
      37       1918.51       5793.97  0.000000E+00  0.000000E+00       2573.14  0.000000E+00       2750.88       7047.63  0.000000E+00       12040.1       62.4264       10374.0 -1.000000E+10 -1.000000E+10 -1.000000E+10 -1.000000E+10 -1.000000E+10  0.000000E+00  0.000000E+00  0.000000E+00  0.000000E+00  0.000000E+00  0.000000E+00  0.000000E+00  0.000000E+00  0.000000E+00  0.000000E+00  0.000000E+00  0.000000E+00       Funtag_D
      38  4.140182E+05  3.714551E+05       42563.0       6.06642     0.0228990      0.275634      0.189241      0.234351  8.549291E-04   -0.00151007    0.00826651       1.03120       Opttag_D
      38       78.4437       1.38870       1.00000  0.000000E+00       1.00000  0.000000E+00      0.881961       81.2449       1.00000       32.6680  0.000000E+00     0.0101216  0.000000E+00     0.0101216  0.000000E+00  0.000000E+00    -0.0202433       79.6522      0.868813        1       Scatag_D
      38       1918.42       5794.67  0.000000E+00  0.000000E+00       2574.35  0.000000E+00       2750.67       7046.20  0.000000E+00       12043.0       62.2251       10373.5 -1.000000E+10 -1.000000E+10 -1.000000E+10 -1.000000E+10 -1.000000E+10  0.000000E+00  0.000000E+00  0.000000E+00  0.000000E+00  0.000000E+00  0.000000E+00  0.000000E+00  0.000000E+00  0.000000E+00  0.000000E+00  0.000000E+00  0.000000E+00       Funtag_D
      39  4.140027E+05  3.714330E+05       42569.7       7.74289     0.0239973      0.294570      0.189209      0.234305  9.079606E-04   -0.00138390    0.00826719       1.03135       Opttag_D
      39       78.4842       1.39018       1.00000  0.000000E+00       1.00000  0.000000E+00      0.881961       81.2610       1.00000       32.6725  0.000000E+00    0.00603415  0.000000E+00    0.00603415  0.000000E+00  0.000000E+00    -0.0120683       79.6522      0.868813        1       Scatag_D
      39       1918.74       5796.31  0.000000E+00  0.000000E+00       2575.98  0.000000E+00       2750.58       7046.20  0.000000E+00       12047.1       61.9797       10372.8 -1.000000E+10 -1.000000E+10 -1.000000E+10 -1.000000E+10 -1.000000E+10  0.000000E+00  0.000000E+00  0.000000E+00  0.000000E+00  0.000000E+00  0.000000E+00  0.000000E+00  0.000000E+00  0.000000E+00  0.000000E+00  0.000000E+00  0.000000E+00       Funtag_D
      40  4.139880E+05  3.713984E+05       42589.6       12.4180     0.0260158      0.329324      0.189155      0.234229  9.908619E-04   -0.00118466    0.00827298       1.03167       Opttag_D
      40       78.5564       1.39289       1.00000  0.000000E+00       1.00000  0.000000E+00      0.881960       81.2893       1.00000       32.6820  0.000000E+00   -0.00126589  0.000000E+00   -0.00126589  0.000000E+00  0.000000E+00    0.00253179       79.6522      0.868813        1       Scatag_D
      40       1921.43       5799.92  0.000000E+00  0.000000E+00       2578.70  0.000000E+00       2750.63       7050.45  0.000000E+00       12055.2       61.5902       10371.6 -1.000000E+10 -1.000000E+10 -1.000000E+10 -1.000000E+10 -1.000000E+10  0.000000E+00  0.000000E+00  0.000000E+00  0.000000E+00  0.000000E+00  0.000000E+00  0.000000E+00  0.000000E+00  0.000000E+00  0.000000E+00  0.000000E+00  0.000000E+00       Funtag_D
      41  4.139693E+05  3.713877E+05       42581.6       7.14897     0.0265950      0.339851      0.189141      0.234200    0.00101657   -0.00110959    0.00827236       1.03139       Opttag_D
      41       78.5763       1.39362       1.00000  0.000000E+00       1.00000  0.000000E+00      0.881962       81.2954       1.00000       32.6848  0.000000E+00   -0.00315899  0.000000E+00   -0.00315899  0.000000E+00  0.000000E+00    0.00631798       79.6522      0.868813        1       Scatag_D
      41       1921.14       5796.81  0.000000E+00  0.000000E+00       2579.38  0.000000E+00       2750.73       7043.53  0.000000E+00       12057.5       61.4760       10371.0 -1.000000E+10 -1.000000E+10 -1.000000E+10 -1.000000E+10 -1.000000E+10  0.000000E+00  0.000000E+00  0.000000E+00  0.000000E+00  0.000000E+00  0.000000E+00  0.000000E+00  0.000000E+00  0.000000E+00  0.000000E+00  0.000000E+00  0.000000E+00       Funtag_D
      42  4.139533E+05  3.713758E+05       42577.5       6.35779     0.0272206      0.351725      0.189125      0.234171    0.00104509   -0.00104378    0.00827398       1.03132       Opttag_D
      42       78.5968       1.39434       1.00000  0.000000E+00       1.00000  0.000000E+00      0.881965       81.2997       1.00000       32.6877  0.000000E+00   -0.00517023  0.000000E+00   -0.00517023  0.000000E+00  0.000000E+00     0.0103405       79.6522      0.868813        1       Scatag_D
      42       1921.89       5796.02  0.000000E+00  0.000000E+00       2580.06  0.000000E+00       2750.71       7037.35  0.000000E+00       12059.9       61.4085       10370.2 -1.000000E+10 -1.000000E+10 -1.000000E+10 -1.000000E+10 -1.000000E+10  0.000000E+00  0.000000E+00  0.000000E+00  0.000000E+00  0.000000E+00  0.000000E+00  0.000000E+00  0.000000E+00  0.000000E+00  0.000000E+00  0.000000E+00  0.000000E+00       Funtag_D
      43  4.139375E+05  3.713574E+05       42580.2       7.51110     0.0282578      0.370921      0.189099      0.234131    0.00108930 -9.678816E-04    0.00827563       1.03136       Opttag_D
      43       78.6305       1.39551       1.00000  0.000000E+00       1.00000  0.000000E+00      0.881971       81.3059       1.00000       32.6931  0.000000E+00   -0.00855223  0.000000E+00   -0.00855223  0.000000E+00  0.000000E+00     0.0171045       79.6522      0.868813        1       Scatag_D
      43       1922.66       5796.46  0.000000E+00  0.000000E+00       2581.09  0.000000E+00       2750.82       7034.48  0.000000E+00       12063.9       61.3836       10369.4 -1.000000E+10 -1.000000E+10 -1.000000E+10 -1.000000E+10 -1.000000E+10  0.000000E+00  0.000000E+00  0.000000E+00  0.000000E+00  0.000000E+00  0.000000E+00  0.000000E+00  0.000000E+00  0.000000E+00  0.000000E+00  0.000000E+00  0.000000E+00       Funtag_D
      44  4.139253E+05  3.713196E+05       42605.6       15.1242     0.0306736      0.415156      0.189044      0.234058    0.00117983 -8.375851E-04    0.00828264       1.03183       Opttag_D
      44       78.7072       1.39826       1.00000  0.000000E+00       1.00000  0.000000E+00      0.881989       81.3173       1.00000       32.7068  0.000000E+00    -0.0161255  0.000000E+00    -0.0161255  0.000000E+00  0.000000E+00     0.0322510       79.6522      0.868813        1       Scatag_D
      44       1925.92       5801.74  0.000000E+00  0.000000E+00       2583.08  0.000000E+00       2752.27       7039.91  0.000000E+00       12073.2       61.0695       10368.4 -1.000000E+10 -1.000000E+10 -1.000000E+10 -1.000000E+10 -1.000000E+10  0.000000E+00  0.000000E+00  0.000000E+00  0.000000E+00  0.000000E+00  0.000000E+00  0.000000E+00  0.000000E+00  0.000000E+00  0.000000E+00  0.000000E+00  0.000000E+00       Funtag_D
      45  4.139020E+05  3.713121E+05       42589.9       7.16885     0.0310473      0.422375      0.189035      0.234053    0.00119776 -8.494903E-04    0.00827689       1.03131       Opttag_D
      45       78.7183       1.39859       1.00000  0.000000E+00       1.00000  0.000000E+00      0.881993       81.3162       1.00000       32.7091  0.000000E+00    -0.0173041  0.000000E+00    -0.0173041  0.000000E+00  0.000000E+00     0.0346081       79.6522      0.868813        1       Scatag_D
      45       1923.25       5795.94  0.000000E+00  0.000000E+00       2583.31  0.000000E+00       2752.64       7031.61  0.000000E+00       12074.3       60.9900       10367.9 -1.000000E+10 -1.000000E+10 -1.000000E+10 -1.000000E+10 -1.000000E+10  0.000000E+00  0.000000E+00  0.000000E+00  0.000000E+00  0.000000E+00  0.000000E+00  0.000000E+00  0.000000E+00  0.000000E+00  0.000000E+00  0.000000E+00  0.000000E+00       Funtag_D
      46  4.138890E+05  3.713031E+05       42585.8       5.95880     0.0314540      0.430405      0.189023      0.234048    0.00121930 -8.623050E-04    0.00827431       1.03127       Opttag_D
      46       78.7301       1.39892       1.00000  0.000000E+00       1.00000  0.000000E+00      0.882000       81.3141       1.00000       32.7112  0.000000E+00    -0.0185024  0.000000E+00    -0.0185024  0.000000E+00  0.000000E+00     0.0370047       79.6522      0.868813        1       Scatag_D
      46       1922.05       5795.42  0.000000E+00  0.000000E+00       2583.51  0.000000E+00       2753.27       7028.15  0.000000E+00       12075.0       60.8333       10367.6 -1.000000E+10 -1.000000E+10 -1.000000E+10 -1.000000E+10 -1.000000E+10  0.000000E+00  0.000000E+00  0.000000E+00  0.000000E+00  0.000000E+00  0.000000E+00  0.000000E+00  0.000000E+00  0.000000E+00  0.000000E+00  0.000000E+00  0.000000E+00       Funtag_D
      47  4.138723E+05  3.712831E+05       42589.2       7.33572     0.0326066      0.452286      0.188996      0.234024    0.00126746 -8.461006E-04    0.00827189       1.03128       Opttag_D
      47       78.7637       1.40004       1.00000  0.000000E+00       1.00000  0.000000E+00      0.882015       81.3135       1.00000       32.7173  0.000000E+00    -0.0217304  0.000000E+00    -0.0217304  0.000000E+00  0.000000E+00     0.0434607       79.6522      0.868813        1       Scatag_D
      47       1920.92       5795.63  0.000000E+00  0.000000E+00       2584.08  0.000000E+00       2755.04       7027.93  0.000000E+00       12077.9       60.5907       10367.2 -1.000000E+10 -1.000000E+10 -1.000000E+10 -1.000000E+10 -1.000000E+10  0.000000E+00  0.000000E+00  0.000000E+00  0.000000E+00  0.000000E+00  0.000000E+00  0.000000E+00  0.000000E+00  0.000000E+00  0.000000E+00  0.000000E+00  0.000000E+00       Funtag_D
      48  4.138525E+05  3.712514E+05       42601.1       13.3577     0.0347826      0.493595      0.188951      0.233979    0.00134330 -7.967434E-04    0.00827200       1.03139       Opttag_D
      48       78.8241       1.40221       1.00000  0.000000E+00       1.00000  0.000000E+00      0.882043       81.3130       1.00000       32.7278  0.000000E+00    -0.0274913  0.000000E+00    -0.0274913  0.000000E+00  0.000000E+00     0.0549827       79.6522      0.868813        1       Scatag_D
      48       1920.98       5796.84  0.000000E+00  0.000000E+00       2584.89  0.000000E+00       2758.79       7029.97  0.000000E+00       12083.3       60.1420       10366.1 -1.000000E+10 -1.000000E+10 -1.000000E+10 -1.000000E+10 -1.000000E+10  0.000000E+00  0.000000E+00  0.000000E+00  0.000000E+00  0.000000E+00  0.000000E+00  0.000000E+00  0.000000E+00  0.000000E+00  0.000000E+00  0.000000E+00  0.000000E+00       Funtag_D
      49  4.138282E+05  3.712296E+05       42598.6       8.32589     0.0365199      0.525794      0.188917      0.233940    0.00139569 -7.501971E-04    0.00827083       1.03101       Opttag_D
      49       78.8712       1.40401       1.00000  0.000000E+00       1.00000  0.000000E+00      0.882064       81.3137       1.00000       32.7369  0.000000E+00    -0.0317615  0.000000E+00    -0.0317615  0.000000E+00  0.000000E+00     0.0635230       79.6522      0.868813        1       Scatag_D
      49       1920.43       5792.55  0.000000E+00  0.000000E+00       2585.48  0.000000E+00       2761.90       7024.60  0.000000E+00       12088.4       59.9375       10365.3 -1.000000E+10 -1.000000E+10 -1.000000E+10 -1.000000E+10 -1.000000E+10  0.000000E+00  0.000000E+00  0.000000E+00  0.000000E+00  0.000000E+00  0.000000E+00  0.000000E+00  0.000000E+00  0.000000E+00  0.000000E+00  0.000000E+00  0.000000E+00       Funtag_D
      50  4.138079E+05  3.712182E+05       42589.7       6.46124     0.0374678      0.543956      0.188901      0.233917    0.00142309 -7.038162E-04    0.00827027       1.03078       Opttag_D
      50       78.8949       1.40494       1.00000  0.000000E+00       1.00000  0.000000E+00      0.882078       81.3127       1.00000       32.7406  0.000000E+00    -0.0338097  0.000000E+00    -0.0338097  0.000000E+00  0.000000E+00     0.0676194       79.6522      0.868813        1       Scatag_D
      50       1920.17       5789.92  0.000000E+00  0.000000E+00       2585.76  0.000000E+00       2763.24       7015.89  0.000000E+00       12090.8       59.8971       10364.1 -1.000000E+10 -1.000000E+10 -1.000000E+10 -1.000000E+10 -1.000000E+10  0.000000E+00  0.000000E+00  0.000000E+00  0.000000E+00  0.000000E+00  0.000000E+00  0.000000E+00  0.000000E+00  0.000000E+00  0.000000E+00  0.000000E+00  0.000000E+00       Funtag_D
      51  4.137865E+05  3.712013E+05       42585.2       8.21280     0.0391541      0.584183      0.188872      0.233880    0.00146356 -6.495811E-04    0.00827119       1.03058       Opttag_D
      51       78.9356       1.40660       1.00000  0.000000E+00       1.00000  0.000000E+00      0.882101       81.3106       1.00000       32.7470  0.000000E+00    -0.0375061  0.000000E+00    -0.0375061  0.000000E+00  0.000000E+00     0.0750122       79.6522      0.868813        1       Scatag_D
      51       1920.60       5787.70  0.000000E+00  0.000000E+00       2586.23  0.000000E+00       2765.22       7008.09  0.000000E+00       12095.5       59.9251       10362.0 -1.000000E+10 -1.000000E+10 -1.000000E+10 -1.000000E+10 -1.000000E+10  0.000000E+00  0.000000E+00  0.000000E+00  0.000000E+00  0.000000E+00  0.000000E+00  0.000000E+00  0.000000E+00  0.000000E+00  0.000000E+00  0.000000E+00  0.000000E+00       Funtag_D
      52  4.137692E+05  3.711850E+05       42584.2       11.7803     0.0413523      0.640957      0.188836      0.233846    0.00150260 -6.025388E-04    0.00827377       1.03022       Opttag_D
      52       78.9864       1.40878       1.00000  0.000000E+00       1.00000  0.000000E+00      0.882131       81.3071       1.00000       32.7555  0.000000E+00    -0.0421557  0.000000E+00    -0.0421557  0.000000E+00  0.000000E+00     0.0843113       79.6522      0.868813        1       Scatag_D
      52       1921.80       5783.64  0.000000E+00  0.000000E+00       2587.10  0.000000E+00       2767.12       7003.29  0.000000E+00       12102.1       60.0129       10359.1 -1.000000E+10 -1.000000E+10 -1.000000E+10 -1.000000E+10 -1.000000E+10  0.000000E+00  0.000000E+00  0.000000E+00  0.000000E+00  0.000000E+00  0.000000E+00  0.000000E+00  0.000000E+00  0.000000E+00  0.000000E+00  0.000000E+00  0.000000E+00       Funtag_D
      53  4.137487E+05  3.711828E+05       42565.9       7.73558     0.0411919      0.638408      0.188836      0.233853    0.00150776 -6.323019E-04    0.00827160       1.03008       Opttag_D
      53       78.9791       1.40840       1.00000  0.000000E+00       1.00000  0.000000E+00      0.882134       81.3026       1.00000       32.7516  0.000000E+00    -0.0415513  0.000000E+00    -0.0415513  0.000000E+00  0.000000E+00     0.0831027       79.6522      0.868813        1       Scatag_D
      53       1920.79       5782.11  0.000000E+00  0.000000E+00       2586.98  0.000000E+00       2765.09       6992.10  0.000000E+00       12100.6       60.1021       10358.1 -1.000000E+10 -1.000000E+10 -1.000000E+10 -1.000000E+10 -1.000000E+10  0.000000E+00  0.000000E+00  0.000000E+00  0.000000E+00  0.000000E+00  0.000000E+00  0.000000E+00  0.000000E+00  0.000000E+00  0.000000E+00  0.000000E+00  0.000000E+00       Funtag_D
      54  4.137265E+05  3.711693E+05       42557.3       7.58115     0.0427276      0.679332      0.188811      0.233841    0.00154024 -6.779387E-04    0.00826827       1.02982       Opttag_D
      54       79.0129       1.40978       1.00000  0.000000E+00       1.00000  0.000000E+00      0.882155       81.2999       1.00000       32.7571  0.000000E+00    -0.0446330  0.000000E+00    -0.0446330  0.000000E+00  0.000000E+00     0.0892661       79.6522      0.868813        1       Scatag_D
      54       1919.24       5779.19  0.000000E+00  0.000000E+00       2587.84  0.000000E+00       2765.30       6984.46  0.000000E+00       12105.3       60.1085       10355.8 -1.000000E+10 -1.000000E+10 -1.000000E+10 -1.000000E+10 -1.000000E+10  0.000000E+00  0.000000E+00  0.000000E+00  0.000000E+00  0.000000E+00  0.000000E+00  0.000000E+00  0.000000E+00  0.000000E+00  0.000000E+00  0.000000E+00  0.000000E+00       Funtag_D
      55  4.137050E+05  3.711563E+05       42548.7       7.73644     0.0438203      0.708233      0.188790      0.233840    0.00157145 -7.535041E-04    0.00826640       1.02963       Opttag_D
      55       79.0346       1.41057       1.00000  0.000000E+00       1.00000  0.000000E+00      0.882172       81.2965       1.00000       32.7600  0.000000E+00    -0.0466026  0.000000E+00    -0.0466026  0.000000E+00  0.000000E+00     0.0932052       79.6522      0.868813        1       Scatag_D
      55       1918.37       5777.09  0.000000E+00  0.000000E+00       2588.68  0.000000E+00       2764.19       6978.71  0.000000E+00       12107.7       60.0909       10353.9 -1.000000E+10 -1.000000E+10 -1.000000E+10 -1.000000E+10 -1.000000E+10  0.000000E+00  0.000000E+00  0.000000E+00  0.000000E+00  0.000000E+00  0.000000E+00  0.000000E+00  0.000000E+00  0.000000E+00  0.000000E+00  0.000000E+00  0.000000E+00       Funtag_D
      56  4.136869E+05  3.711259E+05       42561.0       14.1671     0.0464298      0.776988      0.188746      0.233840    0.00164419 -8.933083E-04    0.00827213       1.02953       Opttag_D
      56       79.0849       1.41247       1.00000  0.000000E+00       1.00000  0.000000E+00      0.882206       81.2934       1.00000       32.7689  0.000000E+00    -0.0508375  0.000000E+00    -0.0508375  0.000000E+00  0.000000E+00      0.101675       79.6522      0.868813        1       Scatag_D
      56       1921.03       5775.91  0.000000E+00  0.000000E+00       2591.16  0.000000E+00       2763.44       6985.34  0.000000E+00       12113.9       60.0465       10350.2 -1.000000E+10 -1.000000E+10 -1.000000E+10 -1.000000E+10 -1.000000E+10  0.000000E+00  0.000000E+00  0.000000E+00  0.000000E+00  0.000000E+00  0.000000E+00  0.000000E+00  0.000000E+00  0.000000E+00  0.000000E+00  0.000000E+00  0.000000E+00       Funtag_D
      57  4.136569E+05  3.711162E+05       42540.8       7.01715     0.0463928      0.777098      0.188743      0.233844    0.00166763 -9.257171E-04    0.00826496       1.02935       Opttag_D
      57       79.0785       1.41197       1.00000  0.000000E+00       1.00000  0.000000E+00      0.882207       81.2916       1.00000       32.7663  0.000000E+00    -0.0502882  0.000000E+00    -0.0502882  0.000000E+00  0.000000E+00      0.100576       79.6522      0.868813        1       Scatag_D
      57       1917.70       5773.93  0.000000E+00  0.000000E+00       2591.31  0.000000E+00       2761.58       6975.62  0.000000E+00       12110.9       60.0477       10349.7 -1.000000E+10 -1.000000E+10 -1.000000E+10 -1.000000E+10 -1.000000E+10  0.000000E+00  0.000000E+00  0.000000E+00  0.000000E+00  0.000000E+00  0.000000E+00  0.000000E+00  0.000000E+00  0.000000E+00  0.000000E+00  0.000000E+00  0.000000E+00       Funtag_D
      58  4.136402E+05  3.711055E+05       42534.7       6.12668     0.0470012      0.794350      0.188734      0.233845    0.00169326 -9.507678E-04    0.00826360       1.02932       Opttag_D
      58       79.0864       1.41218       1.00000  0.000000E+00       1.00000  0.000000E+00      0.882212       81.2923       1.00000       32.7679  0.000000E+00    -0.0511052  0.000000E+00    -0.0511052  0.000000E+00  0.000000E+00      0.102210       79.6522      0.868813        1       Scatag_D
      58       1917.07       5773.53  0.000000E+00  0.000000E+00       2592.06  0.000000E+00       2761.09       6971.77  0.000000E+00       12110.7       60.0721       10348.4 -1.000000E+10 -1.000000E+10 -1.000000E+10 -1.000000E+10 -1.000000E+10  0.000000E+00  0.000000E+00  0.000000E+00  0.000000E+00  0.000000E+00  0.000000E+00  0.000000E+00  0.000000E+00  0.000000E+00  0.000000E+00  0.000000E+00  0.000000E+00       Funtag_D
      59  4.136136E+05  3.710792E+05       42534.4       8.78865     0.0488047      0.844093      0.188708      0.233850    0.00175618   -0.00101740    0.00826172       1.02929       Opttag_D
      59       79.1105       1.41296       1.00000  0.000000E+00       1.00000  0.000000E+00      0.882227       81.2953       1.00000       32.7748  0.000000E+00    -0.0536179  0.000000E+00    -0.0536179  0.000000E+00  0.000000E+00      0.107236       79.6522      0.868813        1       Scatag_D
      59       1916.20       5773.28  0.000000E+00  0.000000E+00       2594.40  0.000000E+00       2761.12       6972.45  0.000000E+00       12112.0       60.1636       10344.8 -1.000000E+10 -1.000000E+10 -1.000000E+10 -1.000000E+10 -1.000000E+10  0.000000E+00  0.000000E+00  0.000000E+00  0.000000E+00  0.000000E+00  0.000000E+00  0.000000E+00  0.000000E+00  0.000000E+00  0.000000E+00  0.000000E+00  0.000000E+00       Funtag_D
      60  4.136054E+05  3.710598E+05       42545.6       15.7983     0.0499043      0.874129      0.188696      0.233886    0.00180279   -0.00110264    0.00826634       1.02945       Opttag_D
      60       79.1173       1.41300       1.00000  0.000000E+00       1.00000  0.000000E+00      0.882229       81.2985       1.00000       32.7798  0.000000E+00    -0.0548897  0.000000E+00    -0.0548897  0.000000E+00  0.000000E+00      0.109779       79.6522      0.868813        1       Scatag_D
      60       1918.34       5775.03  0.000000E+00  0.000000E+00       2595.95  0.000000E+00       2762.14       6981.82  0.000000E+00       12110.1       60.3216       10341.9 -1.000000E+10 -1.000000E+10 -1.000000E+10 -1.000000E+10 -1.000000E+10  0.000000E+00  0.000000E+00  0.000000E+00  0.000000E+00  0.000000E+00  0.000000E+00  0.000000E+00  0.000000E+00  0.000000E+00  0.000000E+00  0.000000E+00  0.000000E+00       Funtag_D
      61  4.135819E+05  3.710578E+05       42524.1       7.24092     0.0502111      0.882253      0.188692      0.233887    0.00180745   -0.00111951    0.00826190       1.02912       Opttag_D
      61       79.1234       1.41324       1.00000  0.000000E+00       1.00000  0.000000E+00      0.882234       81.2976       1.00000       32.7813  0.000000E+00    -0.0553628  0.000000E+00    -0.0553628  0.000000E+00  0.000000E+00      0.110726       79.6522      0.868813        1       Scatag_D
      61       1916.29       5771.28  0.000000E+00  0.000000E+00       2596.39  0.000000E+00       2762.50       6964.66  0.000000E+00       12111.7       60.3079       10341.0 -1.000000E+10 -1.000000E+10 -1.000000E+10 -1.000000E+10 -1.000000E+10  0.000000E+00  0.000000E+00  0.000000E+00  0.000000E+00  0.000000E+00  0.000000E+00  0.000000E+00  0.000000E+00  0.000000E+00  0.000000E+00  0.000000E+00  0.000000E+00       Funtag_D
      62  4.135665E+05  3.710528E+05       42513.7       5.46616     0.0504084      0.887565      0.188688      0.233902    0.00181959   -0.00114754    0.00826263       1.02896       Opttag_D
      62       79.1240       1.41313       1.00000  0.000000E+00       1.00000  0.000000E+00      0.882236       81.2958       1.00000       32.7822  0.000000E+00    -0.0554853  0.000000E+00    -0.0554853  0.000000E+00  0.000000E+00      0.110971       79.6522      0.868813        1       Scatag_D
      62       1916.63       5769.58  0.000000E+00  0.000000E+00       2596.68  0.000000E+00       2763.21       6956.29  0.000000E+00       12111.5       60.3307       10339.5 -1.000000E+10 -1.000000E+10 -1.000000E+10 -1.000000E+10 -1.000000E+10  0.000000E+00  0.000000E+00  0.000000E+00  0.000000E+00  0.000000E+00  0.000000E+00  0.000000E+00  0.000000E+00  0.000000E+00  0.000000E+00  0.000000E+00  0.000000E+00       Funtag_D
      63  4.135499E+05  3.710399E+05       42510.0       7.41839     0.0512219      0.911308      0.188678      0.233941    0.00185052   -0.00122351    0.00826570       1.02881       Opttag_D
      63       79.1287       1.41310       1.00000  0.000000E+00       1.00000  0.000000E+00      0.882241       81.2957       1.00000       32.7854  0.000000E+00    -0.0561588  0.000000E+00    -0.0561588  0.000000E+00  0.000000E+00      0.112318       79.6522      0.868813        1       Scatag_D
      63       1918.05       5767.83  0.000000E+00  0.000000E+00       2597.88  0.000000E+00       2764.79       6953.02  0.000000E+00       12111.5       60.4245       10336.5 -1.000000E+10 -1.000000E+10 -1.000000E+10 -1.000000E+10 -1.000000E+10  0.000000E+00  0.000000E+00  0.000000E+00  0.000000E+00  0.000000E+00  0.000000E+00  0.000000E+00  0.000000E+00  0.000000E+00  0.000000E+00  0.000000E+00  0.000000E+00       Funtag_D
      64  4.135248E+05  3.710196E+05       42505.1       8.74230     0.0521621      0.938742      0.188666      0.234006    0.00189903   -0.00134430    0.00827252       1.02850       Opttag_D
      64       79.1282       1.41263       1.00000  0.000000E+00       1.00000  0.000000E+00      0.882244       81.2939       1.00000       32.7891  0.000000E+00    -0.0563699  0.000000E+00    -0.0563699  0.000000E+00  0.000000E+00      0.112740       79.6522      0.868813        1       Scatag_D
      64       1921.21       5764.35  0.000000E+00  0.000000E+00       2599.21  0.000000E+00       2767.27       6949.92  0.000000E+00       12110.1       60.5874       10332.5 -1.000000E+10 -1.000000E+10 -1.000000E+10 -1.000000E+10 -1.000000E+10  0.000000E+00  0.000000E+00  0.000000E+00  0.000000E+00  0.000000E+00  0.000000E+00  0.000000E+00  0.000000E+00  0.000000E+00  0.000000E+00  0.000000E+00  0.000000E+00       Funtag_D
      65  4.135080E+05  3.710022E+05       42505.8       10.8442     0.0530908      0.966994      0.188656      0.234070    0.00194077   -0.00146286    0.00827986       1.02829       Opttag_D
      65       79.1262       1.41214       1.00000  0.000000E+00       1.00000  0.000000E+00      0.882244       81.2935       1.00000       32.7933  0.000000E+00    -0.0563571  0.000000E+00    -0.0563571  0.000000E+00  0.000000E+00      0.112714       79.6522      0.868813        2       Scatag_D
      65       1924.63       5761.98  0.000000E+00  0.000000E+00       2600.42  0.000000E+00       2769.56       6950.16  0.000000E+00       12109.1       60.7701       10329.2 -1.000000E+10 -1.000000E+10 -1.000000E+10 -1.000000E+10 -1.000000E+10  0.000000E+00  0.000000E+00  0.000000E+00  0.000000E+00  0.000000E+00  0.000000E+00  0.000000E+00  0.000000E+00  0.000000E+00  0.000000E+00  0.000000E+00  0.000000E+00       Funtag_D
      66  4.134851E+05  3.709878E+05       42497.3       6.60715     0.0536053      0.983708      0.188650      0.234120    0.00197537   -0.00154760    0.00828451       1.02799       Opttag_D
      66       79.1193       1.41146       1.00000  0.000000E+00       1.00000  0.000000E+00      0.882242       81.2910       1.00000       32.7957  0.000000E+00    -0.0557606  0.000000E+00    -0.0557606  0.000000E+00  0.000000E+00      0.111521       79.6522      0.868813        1       Scatag_D
      66       1926.79       5758.63  0.000000E+00  0.000000E+00       2600.89  0.000000E+00       2770.89       6944.69  0.000000E+00       12107.3       60.9231       10327.1 -1.000000E+10 -1.000000E+10 -1.000000E+10 -1.000000E+10 -1.000000E+10  0.000000E+00  0.000000E+00  0.000000E+00  0.000000E+00  0.000000E+00  0.000000E+00  0.000000E+00  0.000000E+00  0.000000E+00  0.000000E+00  0.000000E+00  0.000000E+00       Funtag_D
      67  4.134634E+05  3.709711E+05       42492.3       6.36234     0.0541403       1.00291      0.188646      0.234170    0.00201541   -0.00163449    0.00828732       1.02786       Opttag_D
      67       79.1089       1.41055       1.00000  0.000000E+00       1.00000  0.000000E+00      0.882239       81.2872       1.00000       32.7983  0.000000E+00    -0.0547166  0.000000E+00    -0.0547166  0.000000E+00  0.000000E+00      0.109433       79.6522      0.868813        1       Scatag_D
      67       1928.10       5757.18  0.000000E+00  0.000000E+00       2601.22  0.000000E+00       2771.12       6943.24  0.000000E+00       12105.4       61.1168       10325.0 -1.000000E+10 -1.000000E+10 -1.000000E+10 -1.000000E+10 -1.000000E+10  0.000000E+00  0.000000E+00  0.000000E+00  0.000000E+00  0.000000E+00  0.000000E+00  0.000000E+00  0.000000E+00  0.000000E+00  0.000000E+00  0.000000E+00  0.000000E+00       Funtag_D
      68  4.134444E+05  3.709494E+05       42495.0       11.0147     0.0550512       1.03670      0.188641      0.234230    0.00206738   -0.00174958    0.00829154       1.02778       Opttag_D
      68       79.0938       1.40930       1.00000  0.000000E+00       1.00000  0.000000E+00      0.882234       81.2797       1.00000       32.8038  0.000000E+00    -0.0530708  0.000000E+00    -0.0530708  0.000000E+00  0.000000E+00      0.106142       79.6522      0.868813        1       Scatag_D
      68       1930.06       5756.34  0.000000E+00  0.000000E+00       2601.62  0.000000E+00       2770.08       6949.12  0.000000E+00       12104.0       61.4002       10322.4 -1.000000E+10 -1.000000E+10 -1.000000E+10 -1.000000E+10 -1.000000E+10  0.000000E+00  0.000000E+00  0.000000E+00  0.000000E+00  0.000000E+00  0.000000E+00  0.000000E+00  0.000000E+00  0.000000E+00  0.000000E+00  0.000000E+00  0.000000E+00       Funtag_D
      69  4.134225E+05  3.709391E+05       42483.4       7.19502     0.0554449       1.05267      0.188639      0.234254    0.00209202   -0.00181431    0.00828830       1.02779       Opttag_D
      69       79.0840       1.40855       1.00000  0.000000E+00       1.00000  0.000000E+00      0.882229       81.2734       1.00000       32.8062  0.000000E+00    -0.0519010  0.000000E+00    -0.0519010  0.000000E+00  0.000000E+00      0.103802       79.6522      0.868813        1       Scatag_D
      69       1928.55       5756.37  0.000000E+00  0.000000E+00       2601.65  0.000000E+00       2767.89       6942.65  0.000000E+00       12103.6       61.5308       10321.1 -1.000000E+10 -1.000000E+10 -1.000000E+10 -1.000000E+10 -1.000000E+10  0.000000E+00  0.000000E+00  0.000000E+00  0.000000E+00  0.000000E+00  0.000000E+00  0.000000E+00  0.000000E+00  0.000000E+00  0.000000E+00  0.000000E+00  0.000000E+00       Funtag_D
      70  4.134045E+05  3.709319E+05       42472.6       6.00534     0.0557277       1.06415      0.188639      0.234264    0.00210939   -0.00185838    0.00828413       1.02783       Opttag_D
      70       79.0764       1.40796       1.00000  0.000000E+00       1.00000  0.000000E+00      0.882226       81.2658       1.00000       32.8076  0.000000E+00    -0.0508872  0.000000E+00    -0.0508872  0.000000E+00  0.000000E+00      0.101774       79.6522      0.868813        1       Scatag_D
      70       1926.61       5756.86  0.000000E+00  0.000000E+00       2601.63  0.000000E+00       2765.01       6937.03  0.000000E+00       12103.8       61.5700       10320.1 -1.000000E+10 -1.000000E+10 -1.000000E+10 -1.000000E+10 -1.000000E+10  0.000000E+00  0.000000E+00  0.000000E+00  0.000000E+00  0.000000E+00  0.000000E+00  0.000000E+00  0.000000E+00  0.000000E+00  0.000000E+00  0.000000E+00  0.000000E+00       Funtag_D
      71  4.133856E+05  3.709213E+05       42464.3       7.58710     0.0563237       1.08754      0.188639      0.234286    0.00213467   -0.00194725    0.00828236       1.02794       Opttag_D
      71       79.0638       1.40701       1.00000  0.000000E+00       1.00000  0.000000E+00      0.882222       81.2537       1.00000       32.8108  0.000000E+00    -0.0491608  0.000000E+00    -0.0491608  0.000000E+00  0.000000E+00     0.0983216       79.6522      0.868813        1       Scatag_D
      71       1925.79       5758.11  0.000000E+00  0.000000E+00       2601.64  0.000000E+00       2761.57       6933.07  0.000000E+00       12104.5       61.5696       10318.0 -1.000000E+10 -1.000000E+10 -1.000000E+10 -1.000000E+10 -1.000000E+10  0.000000E+00  0.000000E+00  0.000000E+00  0.000000E+00  0.000000E+00  0.000000E+00  0.000000E+00  0.000000E+00  0.000000E+00  0.000000E+00  0.000000E+00  0.000000E+00       Funtag_D
      72  4.133701E+05  3.709110E+05       42459.1       9.66882     0.0571684       1.11924      0.188639      0.234325    0.00215946   -0.00210359    0.00828513       1.02795       Opttag_D
      72       79.0470       1.40580       1.00000  0.000000E+00       1.00000  0.000000E+00      0.882215       81.2373       1.00000       32.8156  0.000000E+00    -0.0466821  0.000000E+00    -0.0466821  0.000000E+00  0.000000E+00     0.0933642       79.6522      0.868813        1       Scatag_D
      72       1927.07       5758.18  0.000000E+00  0.000000E+00       2601.58  0.000000E+00       2758.13       6932.11  0.000000E+00       12106.0       61.4761       10314.6 -1.000000E+10 -1.000000E+10 -1.000000E+10 -1.000000E+10 -1.000000E+10  0.000000E+00  0.000000E+00  0.000000E+00  0.000000E+00  0.000000E+00  0.000000E+00  0.000000E+00  0.000000E+00  0.000000E+00  0.000000E+00  0.000000E+00  0.000000E+00       Funtag_D
      73  4.133544E+05  3.709086E+05       42445.8       5.90406     0.0573294       1.12365      0.188640      0.234330    0.00216525   -0.00213319    0.00828351       1.02780       Opttag_D
      73       79.0438       1.40554       1.00000  0.000000E+00       1.00000  0.000000E+00      0.882214       81.2334       1.00000       32.8168  0.000000E+00    -0.0461998  0.000000E+00    -0.0461998  0.000000E+00  0.000000E+00     0.0923995       79.6522      0.868813        1       Scatag_D
      73       1926.32       5756.52  0.000000E+00  0.000000E+00       2601.55  0.000000E+00       2758.02       6923.27  0.000000E+00       12105.8       61.3759       10313.0 -1.000000E+10 -1.000000E+10 -1.000000E+10 -1.000000E+10 -1.000000E+10  0.000000E+00  0.000000E+00  0.000000E+00  0.000000E+00  0.000000E+00  0.000000E+00  0.000000E+00  0.000000E+00  0.000000E+00  0.000000E+00  0.000000E+00  0.000000E+00       Funtag_D
      74  4.133382E+05  3.709032E+05       42435.1       5.63626     0.0577245       1.13589      0.188639      0.234348    0.00217817   -0.00221107    0.00828338       1.02762       Opttag_D
      74       79.0363       1.40497       1.00000  0.000000E+00       1.00000  0.000000E+00      0.882210       81.2267       1.00000       32.8199  0.000000E+00    -0.0451003  0.000000E+00    -0.0451003  0.000000E+00  0.000000E+00     0.0902007       79.6522      0.868813        1       Scatag_D
      74       1926.26       5754.53  0.000000E+00  0.000000E+00       2601.47  0.000000E+00       2758.18       6918.24  0.000000E+00       12105.5       61.2403       10309.6 -1.000000E+10 -1.000000E+10 -1.000000E+10 -1.000000E+10 -1.000000E+10  0.000000E+00  0.000000E+00  0.000000E+00  0.000000E+00  0.000000E+00  0.000000E+00  0.000000E+00  0.000000E+00  0.000000E+00  0.000000E+00  0.000000E+00  0.000000E+00       Funtag_D
      75  4.133232E+05  3.708951E+05       42428.1       6.73722     0.0583528       1.15708      0.188640      0.234375    0.00219759   -0.00232756    0.00828453       1.02748       Opttag_D
      75       79.0248       1.40413       1.00000  0.000000E+00       1.00000  0.000000E+00      0.882203       81.2175       1.00000       32.8252  0.000000E+00    -0.0434631  0.000000E+00    -0.0434631  0.000000E+00  0.000000E+00     0.0869262       79.6522      0.868813        1       Scatag_D
      75       1926.80       5752.89  0.000000E+00  0.000000E+00       2601.43  0.000000E+00       2758.23       6917.43  0.000000E+00       12105.8       61.1520       10304.4 -1.000000E+10 -1.000000E+10 -1.000000E+10 -1.000000E+10 -1.000000E+10  0.000000E+00  0.000000E+00  0.000000E+00  0.000000E+00  0.000000E+00  0.000000E+00  0.000000E+00  0.000000E+00  0.000000E+00  0.000000E+00  0.000000E+00  0.000000E+00       Funtag_D
      76  4.133039E+05  3.708823E+05       42421.6       11.3474     0.0599274       1.20768      0.188648      0.234441    0.00222831   -0.00261858    0.00829078       1.02730       Opttag_D
      76       78.9980       1.40231       1.00000  0.000000E+00       1.00000  0.000000E+00      0.882183       81.1997       1.00000       32.8399  0.000000E+00    -0.0397178  0.000000E+00    -0.0397178  0.000000E+00  0.000000E+00     0.0794356       79.6522      0.868813        1       Scatag_D
      76       1929.71       5750.92  0.000000E+00  0.000000E+00       2601.45  0.000000E+00       2758.97       6924.66  0.000000E+00       12107.5       61.1010       10287.3 -1.000000E+10 -1.000000E+10 -1.000000E+10 -1.000000E+10 -1.000000E+10  0.000000E+00  0.000000E+00  0.000000E+00  0.000000E+00  0.000000E+00  0.000000E+00  0.000000E+00  0.000000E+00  0.000000E+00  0.000000E+00  0.000000E+00  0.000000E+00       Funtag_D
      77  4.132810E+05  3.708755E+05       42405.5       6.82629     0.0605014       1.22302      0.188650      0.234464    0.00224462   -0.00272713    0.00828897       1.02712       Opttag_D
      77       78.9887       1.40160       1.00000  0.000000E+00       1.00000  0.000000E+00      0.882175       81.1936       1.00000       32.8467  0.000000E+00    -0.0385375  0.000000E+00    -0.0385375  0.000000E+00  0.000000E+00     0.0770751       79.6522      0.868813        1       Scatag_D
      77       1928.86       5748.88  0.000000E+00  0.000000E+00       2601.51  0.000000E+00       2759.51       6919.83  0.000000E+00       12108.1       61.1562       10277.7 -1.000000E+10 -1.000000E+10 -1.000000E+10 -1.000000E+10 -1.000000E+10  0.000000E+00  0.000000E+00  0.000000E+00  0.000000E+00  0.000000E+00  0.000000E+00  0.000000E+00  0.000000E+00  0.000000E+00  0.000000E+00  0.000000E+00  0.000000E+00       Funtag_D
      78  4.132593E+05  3.708673E+05       42392.0       7.16763     0.0612509       1.24140      0.188652      0.234492    0.00226425   -0.00286264    0.00828848       1.02710       Opttag_D
      78       78.9773       1.40074       1.00000  0.000000E+00       1.00000  0.000000E+00      0.882161       81.1887       1.00000       32.8554  0.000000E+00    -0.0371817  0.000000E+00    -0.0371817  0.000000E+00  0.000000E+00     0.0743633       79.6522      0.868813        1       Scatag_D
      78       1928.63       5748.68  0.000000E+00  0.000000E+00       2601.62  0.000000E+00       2760.28       6921.45  0.000000E+00       12108.4       61.2727       10261.6 -1.000000E+10 -1.000000E+10 -1.000000E+10 -1.000000E+10 -1.000000E+10  0.000000E+00  0.000000E+00  0.000000E+00  0.000000E+00  0.000000E+00  0.000000E+00  0.000000E+00  0.000000E+00  0.000000E+00  0.000000E+00  0.000000E+00  0.000000E+00       Funtag_D
      79  4.132358E+05  3.708577E+05       42378.1       8.94497     0.0626620       1.27753      0.188658      0.234545    0.00228727   -0.00311025    0.00829590       1.02743       Opttag_D
      79       78.9584       1.39941       1.00000  0.000000E+00       1.00000  0.000000E+00      0.882137       81.1838       1.00000       32.8707  0.000000E+00    -0.0349799  0.000000E+00    -0.0349799  0.000000E+00  0.000000E+00     0.0699599       79.6522      0.868813        1       Scatag_D
      79       1932.09       5752.38  0.000000E+00  0.000000E+00       2602.12  0.000000E+00       2761.74       6930.82  0.000000E+00       12109.9       61.3505       10227.6 -1.000000E+10 -1.000000E+10 -1.000000E+10 -1.000000E+10 -1.000000E+10  0.000000E+00  0.000000E+00  0.000000E+00  0.000000E+00  0.000000E+00  0.000000E+00  0.000000E+00  0.000000E+00  0.000000E+00  0.000000E+00  0.000000E+00  0.000000E+00       Funtag_D
      80  4.132272E+05  3.708468E+05       42380.4       16.7322     0.0644109       1.30947      0.188665      0.234597    0.00231340   -0.00342433    0.00830386       1.02852       Opttag_D
      80       78.9392       1.39797       1.00000  0.000000E+00       1.00000  0.000000E+00      0.882097       81.1872       1.00000       32.8894  0.000000E+00    -0.0332476  0.000000E+00    -0.0332476  0.000000E+00  0.000000E+00     0.0664951       79.6522      0.868813        1       Scatag_D
      80       1935.80       5764.56  0.000000E+00  0.000000E+00       2603.10  0.000000E+00       2763.42       6955.98  0.000000E+00       12111.2       61.3267       10185.0 -1.000000E+10 -1.000000E+10 -1.000000E+10 -1.000000E+10 -1.000000E+10  0.000000E+00  0.000000E+00  0.000000E+00  0.000000E+00  0.000000E+00  0.000000E+00  0.000000E+00  0.000000E+00  0.000000E+00  0.000000E+00  0.000000E+00  0.000000E+00       Funtag_D
      81  4.131946E+05  3.708397E+05       42354.9       7.61525     0.0632931       1.27739      0.188653      0.234543    0.00233056   -0.00319772    0.00829777       1.02745       Opttag_D
      81       78.9549       1.39871       1.00000  0.000000E+00       1.00000  0.000000E+00      0.882111       81.1958       1.00000       32.8759  0.000000E+00    -0.0356682  0.000000E+00    -0.0356682  0.000000E+00  0.000000E+00     0.0713365       79.6522      0.868813        1       Scatag_D
      81       1932.96       5752.63  0.000000E+00  0.000000E+00       2602.17  0.000000E+00       2761.69       6931.87  0.000000E+00       12108.7       61.2700       10203.6 -1.000000E+10 -1.000000E+10 -1.000000E+10 -1.000000E+10 -1.000000E+10  0.000000E+00  0.000000E+00  0.000000E+00  0.000000E+00  0.000000E+00  0.000000E+00  0.000000E+00  0.000000E+00  0.000000E+00  0.000000E+00  0.000000E+00  0.000000E+00       Funtag_D
      82  4.131798E+05  3.708343E+05       42345.5       5.84207     0.0636441       1.28655      0.188649      0.234547    0.00234327   -0.00324088    0.00829379       1.02752       Opttag_D
      82       78.9523       1.39846       1.00000  0.000000E+00       1.00000  0.000000E+00      0.882107       81.1986       1.00000       32.8779  0.000000E+00    -0.0356667  0.000000E+00    -0.0356667  0.000000E+00  0.000000E+00     0.0713335       79.6522      0.868813        1       Scatag_D
      82       1931.10       5753.37  0.000000E+00  0.000000E+00       2602.34  0.000000E+00       2761.79       6931.09  0.000000E+00       12109.6       61.2208       10195.0 -1.000000E+10 -1.000000E+10 -1.000000E+10 -1.000000E+10 -1.000000E+10  0.000000E+00  0.000000E+00  0.000000E+00  0.000000E+00  0.000000E+00  0.000000E+00  0.000000E+00  0.000000E+00  0.000000E+00  0.000000E+00  0.000000E+00  0.000000E+00       Funtag_D
      83  4.131585E+05  3.708222E+05       42336.3       5.50322     0.0640883       1.29338      0.188643      0.234537    0.00237250   -0.00328213    0.00830035       1.02747       Opttag_D
      83       78.9519       1.39817       1.00000  0.000000E+00       1.00000  0.000000E+00      0.882101       81.2070       1.00000       32.8779  0.000000E+00    -0.0364073  0.000000E+00    -0.0364073  0.000000E+00  0.000000E+00     0.0728146       79.6522      0.868813        1       Scatag_D
      83       1934.16       5752.78  0.000000E+00  0.000000E+00       2602.44  0.000000E+00       2761.53       6931.70  0.000000E+00       12110.9       61.0961       10181.7 -1.000000E+10 -1.000000E+10 -1.000000E+10 -1.000000E+10 -1.000000E+10  0.000000E+00  0.000000E+00  0.000000E+00  0.000000E+00  0.000000E+00  0.000000E+00  0.000000E+00  0.000000E+00  0.000000E+00  0.000000E+00  0.000000E+00  0.000000E+00       Funtag_D
      84  4.131419E+05  3.708081E+05       42333.8       8.59583     0.0646326       1.31207      0.188633      0.234522    0.00240619   -0.00332297    0.00830356       1.02757       Opttag_D
      84       78.9518       1.39781       1.00000  0.000000E+00       1.00000  0.000000E+00      0.882094       81.2119       1.00000       32.8789  0.000000E+00    -0.0373595  0.000000E+00    -0.0373595  0.000000E+00  0.000000E+00     0.0747189       79.6522      0.868813        1       Scatag_D
      84       1935.66       5753.99  0.000000E+00  0.000000E+00       2602.59  0.000000E+00       2761.54       6939.86  0.000000E+00       12113.1       61.0910       10165.9 -1.000000E+10 -1.000000E+10 -1.000000E+10 -1.000000E+10 -1.000000E+10  0.000000E+00  0.000000E+00  0.000000E+00  0.000000E+00  0.000000E+00  0.000000E+00  0.000000E+00  0.000000E+00  0.000000E+00  0.000000E+00  0.000000E+00  0.000000E+00       Funtag_D
      85  4.131245E+05  3.707978E+05       42326.8       6.08775     0.0649315       1.32936      0.188624      0.234501    0.00243090   -0.00332595    0.00830391       1.02715       Opttag_D
      85       78.9544       1.39770       1.00000  0.000000E+00       1.00000  0.000000E+00      0.882090       81.2145       1.00000       32.8771  0.000000E+00    -0.0384178  0.000000E+00    -0.0384178  0.000000E+00  0.000000E+00     0.0768357       79.6522      0.868813        1       Scatag_D
      85       1935.82       5749.29  0.000000E+00  0.000000E+00       2602.59  0.000000E+00       2761.71       6945.14  0.000000E+00       12114.9       61.1823       10156.1 -1.000000E+10 -1.000000E+10 -1.000000E+10 -1.000000E+10 -1.000000E+10  0.000000E+00  0.000000E+00  0.000000E+00  0.000000E+00  0.000000E+00  0.000000E+00  0.000000E+00  0.000000E+00  0.000000E+00  0.000000E+00  0.000000E+00  0.000000E+00       Funtag_D
      86  4.131128E+05  3.707943E+05       42318.5       4.96159     0.0649495       1.32676      0.188620      0.234487    0.00243923   -0.00329544    0.00829835       1.02688       Opttag_D
      86       78.9564       1.39773       1.00000  0.000000E+00       1.00000  0.000000E+00      0.882091       81.2125       1.00000       32.8753  0.000000E+00    -0.0388475  0.000000E+00    -0.0388475  0.000000E+00  0.000000E+00     0.0776951       79.6522      0.868813        1       Scatag_D
      86       1933.23       5746.25  0.000000E+00  0.000000E+00       2602.61  0.000000E+00       2761.73       6944.69  0.000000E+00       12115.3       61.2990       10153.4 -1.000000E+10 -1.000000E+10 -1.000000E+10 -1.000000E+10 -1.000000E+10  0.000000E+00  0.000000E+00  0.000000E+00  0.000000E+00  0.000000E+00  0.000000E+00  0.000000E+00  0.000000E+00  0.000000E+00  0.000000E+00  0.000000E+00  0.000000E+00       Funtag_D
      87  4.130976E+05  3.707837E+05       42313.9       6.61612     0.0652182       1.33201      0.188613      0.234465    0.00246473   -0.00327238    0.00829630       1.02656       Opttag_D
      87       78.9596       1.39767       1.00000  0.000000E+00       1.00000  0.000000E+00      0.882088       81.2102       1.00000       32.8717  0.000000E+00    -0.0398700  0.000000E+00    -0.0398700  0.000000E+00  0.000000E+00     0.0797399       79.6522      0.868813        1       Scatag_D
      87       1932.27       5742.64  0.000000E+00  0.000000E+00       2602.74  0.000000E+00       2762.27       6951.89  0.000000E+00       12117.3       61.4901       10143.3 -1.000000E+10 -1.000000E+10 -1.000000E+10 -1.000000E+10 -1.000000E+10  0.000000E+00  0.000000E+00  0.000000E+00  0.000000E+00  0.000000E+00  0.000000E+00  0.000000E+00  0.000000E+00  0.000000E+00  0.000000E+00  0.000000E+00  0.000000E+00       Funtag_D
      88  4.130873E+05  3.707745E+05       42312.8       7.28048     0.0656196       1.34645      0.188605      0.234443    0.00248669   -0.00325700    0.00829330       1.02655       Opttag_D
      88       78.9622       1.39764       1.00000  0.000000E+00       1.00000  0.000000E+00      0.882084       81.2107       1.00000       32.8684  0.000000E+00    -0.0408908  0.000000E+00    -0.0408908  0.000000E+00  0.000000E+00     0.0817816       79.6522      0.868813        1       Scatag_D
      88       1930.88       5742.48  0.000000E+00  0.000000E+00       2602.86  0.000000E+00       2763.09       6961.35  0.000000E+00       12119.8       61.5691       10130.8 -1.000000E+10 -1.000000E+10 -1.000000E+10 -1.000000E+10 -1.000000E+10  0.000000E+00  0.000000E+00  0.000000E+00  0.000000E+00  0.000000E+00  0.000000E+00  0.000000E+00  0.000000E+00  0.000000E+00  0.000000E+00  0.000000E+00  0.000000E+00       Funtag_D
      89  4.130783E+05  3.707726E+05       42305.7       4.69985     0.0657332       1.35193      0.188603      0.234447    0.00249128   -0.00328122    0.00829165       1.02653       Opttag_D
      89       78.9618       1.39759       1.00000  0.000000E+00       1.00000  0.000000E+00      0.882082       81.2115       1.00000       32.8678  0.000000E+00    -0.0409825  0.000000E+00    -0.0409825  0.000000E+00  0.000000E+00     0.0819651       79.6522      0.868813        1       Scatag_D
      89       1930.11       5742.36  0.000000E+00  0.000000E+00       2602.88  0.000000E+00       2762.99       6958.73  0.000000E+00       12120.2       61.4976       10126.9 -1.000000E+10 -1.000000E+10 -1.000000E+10 -1.000000E+10 -1.000000E+10  0.000000E+00  0.000000E+00  0.000000E+00  0.000000E+00  0.000000E+00  0.000000E+00  0.000000E+00  0.000000E+00  0.000000E+00  0.000000E+00  0.000000E+00  0.000000E+00       Funtag_D
      90  4.130660E+05  3.707680E+05       42298.0       4.10658     0.0661182       1.36919      0.188600      0.234452    0.00250236   -0.00334637    0.00829094       1.02664       Opttag_D
      90       78.9615       1.39752       1.00000  0.000000E+00       1.00000  0.000000E+00      0.882075       81.2133       1.00000       32.8664  0.000000E+00    -0.0413945  0.000000E+00    -0.0413945  0.000000E+00  0.000000E+00     0.0827890       79.6522      0.868813        1       Scatag_D
      90       1929.78       5743.54  0.000000E+00  0.000000E+00       2602.87  0.000000E+00       2763.09       6960.24  0.000000E+00       12121.8       61.3352       10115.4 -1.000000E+10 -1.000000E+10 -1.000000E+10 -1.000000E+10 -1.000000E+10  0.000000E+00  0.000000E+00  0.000000E+00  0.000000E+00  0.000000E+00  0.000000E+00  0.000000E+00  0.000000E+00  0.000000E+00  0.000000E+00  0.000000E+00  0.000000E+00       Funtag_D
      91  4.130575E+05  3.707667E+05       42290.8       5.19685     0.0664343       1.37917      0.188600      0.234454    0.00250547   -0.00339424    0.00828720       1.02666       Opttag_D
      91       78.9623       1.39754       1.00000  0.000000E+00       1.00000  0.000000E+00      0.882067       81.2127       1.00000       32.8653  0.000000E+00    -0.0417928  0.000000E+00    -0.0417928  0.000000E+00  0.000000E+00     0.0835856       79.6522      0.868813        1       Scatag_D
      91       1928.04       5743.72  0.000000E+00  0.000000E+00       2602.85  0.000000E+00       2763.29       6962.97  0.000000E+00       12122.9       61.2776       10105.8 -1.000000E+10 -1.000000E+10 -1.000000E+10 -1.000000E+10 -1.000000E+10  0.000000E+00  0.000000E+00  0.000000E+00  0.000000E+00  0.000000E+00  0.000000E+00  0.000000E+00  0.000000E+00  0.000000E+00  0.000000E+00  0.000000E+00  0.000000E+00       Funtag_D
      92  4.130422E+05  3.707676E+05       42274.6       5.20480     0.0670993       1.39476      0.188600      0.234460    0.00250328   -0.00350403    0.00828169       1.02653       Opttag_D
      92       78.9641       1.39766       1.00000  0.000000E+00       1.00000  0.000000E+00      0.882053       81.2095       1.00000       32.8639  0.000000E+00    -0.0423158  0.000000E+00    -0.0423158  0.000000E+00  0.000000E+00     0.0846315       79.6522      0.868813        1       Scatag_D
      92       1925.48       5742.31  0.000000E+00  0.000000E+00       2602.70  0.000000E+00       2763.99       6967.13  0.000000E+00       12124.7       61.2230       10087.1 -1.000000E+10 -1.000000E+10 -1.000000E+10 -1.000000E+10 -1.000000E+10  0.000000E+00  0.000000E+00  0.000000E+00  0.000000E+00  0.000000E+00  0.000000E+00  0.000000E+00  0.000000E+00  0.000000E+00  0.000000E+00  0.000000E+00  0.000000E+00       Funtag_D
      93  4.130292E+05  3.707731E+05       42256.1       9.28158     0.0678165       1.40130      0.188602      0.234470    0.00249008   -0.00362887    0.00827938       1.02631       Opttag_D
      93       78.9663       1.39790       1.00000  0.000000E+00       1.00000  0.000000E+00      0.882036       81.2061       1.00000       32.8625  0.000000E+00    -0.0425945  0.000000E+00    -0.0425945  0.000000E+00  0.000000E+00     0.0851890       79.6522      0.868813        1       Scatag_D
      93       1924.40       5739.81  0.000000E+00  0.000000E+00       2602.39  0.000000E+00       2764.68       6970.97  0.000000E+00       12125.9       61.2771       10066.7 -1.000000E+10 -1.000000E+10 -1.000000E+10 -1.000000E+10 -1.000000E+10  0.000000E+00  0.000000E+00  0.000000E+00  0.000000E+00  0.000000E+00  0.000000E+00  0.000000E+00  0.000000E+00  0.000000E+00  0.000000E+00  0.000000E+00  0.000000E+00       Funtag_D
      94  4.130123E+05  3.707771E+05       42235.2       5.58146     0.0681708       1.39941      0.188605      0.234474    0.00248054   -0.00369295    0.00827636       1.02609       Opttag_D
      94       78.9675       1.39800       1.00000  0.000000E+00       1.00000  0.000000E+00      0.882030       81.2010       1.00000       32.8623  0.000000E+00    -0.0423605  0.000000E+00    -0.0423605  0.000000E+00  0.000000E+00     0.0847210       79.6522      0.868813        1       Scatag_D
      94       1923.00       5737.35  0.000000E+00  0.000000E+00       2602.17  0.000000E+00       2763.91       6966.20  0.000000E+00       12125.6       61.3681       10055.5 -1.000000E+10 -1.000000E+10 -1.000000E+10 -1.000000E+10 -1.000000E+10  0.000000E+00  0.000000E+00  0.000000E+00  0.000000E+00  0.000000E+00  0.000000E+00  0.000000E+00  0.000000E+00  0.000000E+00  0.000000E+00  0.000000E+00  0.000000E+00       Funtag_D
      95  4.129949E+05  3.707809E+05       42214.0       5.30540     0.0685646       1.39305      0.188608      0.234488    0.00247144   -0.00378397    0.00827605       1.02595       Opttag_D
      95       78.9683       1.39805       1.00000  0.000000E+00       1.00000  0.000000E+00      0.882024       81.1942       1.00000       32.8616  0.000000E+00    -0.0418089  0.000000E+00    -0.0418089  0.000000E+00  0.000000E+00     0.0836178       79.6522      0.868813        1       Scatag_D
      95       1922.86       5735.78  0.000000E+00  0.000000E+00       2601.78  0.000000E+00       2762.16       6963.37  0.000000E+00       12124.4       61.4543       10042.2 -1.000000E+10 -1.000000E+10 -1.000000E+10 -1.000000E+10 -1.000000E+10  0.000000E+00  0.000000E+00  0.000000E+00  0.000000E+00  0.000000E+00  0.000000E+00  0.000000E+00  0.000000E+00  0.000000E+00  0.000000E+00  0.000000E+00  0.000000E+00       Funtag_D
      96  4.129769E+05  3.707858E+05       42191.1       7.68167     0.0693132       1.39093      0.188613      0.234515    0.00245964   -0.00394197    0.00827655       1.02595       Opttag_D
      96       78.9695       1.39811       1.00000  0.000000E+00       1.00000  0.000000E+00      0.882017       81.1839       1.00000       32.8609  0.000000E+00    -0.0412935  0.000000E+00    -0.0412935  0.000000E+00  0.000000E+00     0.0825869       79.6522      0.868813        1       Scatag_D
      96       1923.09       5735.85  0.000000E+00  0.000000E+00       2601.18  0.000000E+00       2760.28       6966.43  0.000000E+00       12123.9       61.4432       10019.0 -1.000000E+10 -1.000000E+10 -1.000000E+10 -1.000000E+10 -1.000000E+10  0.000000E+00  0.000000E+00  0.000000E+00  0.000000E+00  0.000000E+00  0.000000E+00  0.000000E+00  0.000000E+00  0.000000E+00  0.000000E+00  0.000000E+00  0.000000E+00       Funtag_D
      97  4.129502E+05  3.707996E+05       42150.6       8.84985     0.0708652       1.45253      0.188623      0.234577    0.00242652   -0.00429392    0.00827831       1.02644       Opttag_D
      97       78.9714       1.39829       1.00000  0.000000E+00       1.00000  0.000000E+00      0.882005       81.1642       1.00000       32.8617  0.000000E+00    -0.0405541  0.000000E+00    -0.0405541  0.000000E+00  0.000000E+00     0.0811081       79.6522      0.868813        1       Scatag_D
      97       1923.90       5741.29  0.000000E+00  0.000000E+00       2599.97  0.000000E+00       2759.04       6973.24  0.000000E+00       12124.4       61.2244       9967.50 -1.000000E+10 -1.000000E+10 -1.000000E+10 -1.000000E+10 -1.000000E+10  0.000000E+00  0.000000E+00  0.000000E+00  0.000000E+00  0.000000E+00  0.000000E+00  0.000000E+00  0.000000E+00  0.000000E+00  0.000000E+00  0.000000E+00  0.000000E+00       Funtag_D

 An atom has moved more than     1.50 A from the initial/last
 position - if this was an accepted trial point, probably need to update the
 non-bonded contact list.

      98  4.129332E+05  3.708080E+05       42125.2       9.24087     0.0720585       1.57453      0.188629      0.234634    0.00240647   -0.00456608    0.00827767       1.02688       Opttag_D
      98       78.9719       1.39832       1.00000  0.000000E+00       1.00000  0.000000E+00      0.882001       81.1466       1.00000       32.8646  0.000000E+00    -0.0401684  0.000000E+00    -0.0401684  0.000000E+00  0.000000E+00     0.0803369       79.6522      0.868813        1       Scatag_D
      98       1923.61       5746.19  0.000000E+00  0.000000E+00       2599.16  0.000000E+00       2761.00       6975.53  0.000000E+00       12125.0       60.9669       9933.76 -1.000000E+10 -1.000000E+10 -1.000000E+10 -1.000000E+10 -1.000000E+10  0.000000E+00  0.000000E+00  0.000000E+00  0.000000E+00  0.000000E+00  0.000000E+00  0.000000E+00  0.000000E+00  0.000000E+00  0.000000E+00  0.000000E+00  0.000000E+00       Funtag_D

 An atom has moved more than     1.50 A from the initial/last
 position - need to update the non-bonded contact list.


 ############################################################################################################
 ###################################################### setting up contact terms
 ############################################################################################################

      99  4.129067E+05  3.707887E+05       42118.1       9.24652     0.0712493       1.51656      0.188618      0.234625    0.00245279   -0.00443984    0.00828072       1.02654       Opttag_D
      99       78.9686       1.39777       1.00000  0.000000E+00       1.00000  0.000000E+00      0.882014       81.1515       1.00000       32.8626  0.000000E+00    -0.0406638  0.000000E+00    -0.0406638  0.000000E+00  0.000000E+00     0.0813277       79.6522      0.868813        1       Scatag_D
      99       1925.02       5742.42  0.000000E+00  0.000000E+00       2600.05  0.000000E+00       2759.07       6969.21  0.000000E+00       12121.6       60.7431       9939.94 -1.000000E+10 -1.000000E+10 -1.000000E+10 -1.000000E+10 -1.000000E+10  0.000000E+00  0.000000E+00  0.000000E+00  0.000000E+00  0.000000E+00  0.000000E+00  0.000000E+00  0.000000E+00  0.000000E+00  0.000000E+00  0.000000E+00  0.000000E+00       Funtag_D

   MaxLik Scaling Cycle 0106

   Wilson plot

   Statistics in resolution bins after refinement cycle 0003
```


|  |  |
| --- | --- |
|  | - Rxpct factors plot- Rxpct factors table- <Log-Lik Gain>- Ln Sigma A- Correlation Coefficients |


```
   Iters     Tot_Funct    Xray_Funct    Geom_Funct          Grms     rmsD2init     maxD2init             R         Rfree           LLG       LLGfree     rms(bond)    rms(angle)
   Iters             K   B_IMPF_FRAG        K_MISS   B_TEMP_MISS   K_IMPF_MISS   B_IMPF_MISS        K_SOLV   B_TEMP_SOLV   K_IMPF_SOLV   B_IMPF_SOLV        B_TEMP     B_TEMP_11     B_TEMP_12     B_TEMP_22     B_TEMP_13     B_TEMP_23     B_TEMP_33      K_WILSON      B_WILSON      FGCYCLES
   Iters          BOND         ANGLE      IMPROPER       TORSION    SINTORSION      TRIGONAL        PLANAR  BCORRELATION       CONTACT     IDEALDIST        CHIRAL    SIMILARITY       OCCSSUM  UTILDISTANCE     UTILANGLE   UTILTORSION        RHOFIT        ADP-01        ADP-02        ADP-03        ADP-04        ADP-05        ADP-06        ADP-07        ADP-08        ADP-09        ADP-10      RHOFITPC            QM
     100  4.128909E+05  3.707892E+05       42101.7       10.2904     0.0716175       1.57479      0.188621      0.234648    0.00245159   -0.00452508    0.00828300       1.02659       Opttag_D
     100       78.9675       1.39764       1.00000  0.000000E+00       1.00000  0.000000E+00      0.882013       81.1478       1.00000       32.8655  0.000000E+00    -0.0406136  0.000000E+00    -0.0406136  0.000000E+00  0.000000E+00     0.0812273       79.6522      0.868813        1       Scatag_D
     100       1926.09       5742.98  0.000000E+00  0.000000E+00       2599.70  0.000000E+00       2760.48       6966.75  0.000000E+00       12119.5       60.7284       9925.56 -1.000000E+10 -1.000000E+10 -1.000000E+10 -1.000000E+10 -1.000000E+10  0.000000E+00  0.000000E+00  0.000000E+00  0.000000E+00  0.000000E+00  0.000000E+00  0.000000E+00  0.000000E+00  0.000000E+00  0.000000E+00  0.000000E+00  0.000000E+00       Funtag_D
   Geometry Restraint Deviations SCREEN Information

 
 No -bcorrel_zeroocchydrogens_factor flag found - keeping default: will set B for OCC=0 hydrogens to    1.200 times heavy-atom B


 TLS information. Total number of groups:     2


 Group   Natoms    FixRB   FixTLS   Group Tag    Group Spec
     1     2770      Y        Y     tls1    { A|* }
     2     2767      Y        Y     tls2    { B|* }
 
NOTE BUSTER_TLS_SET tls1 { A|* }
NOTE BUSTER_TLS_T   tls1     -1.78214E-01 -9.18952E-02  2.39945E-01 -3.34788E-02  3.82832E-02  7.05171E-03
NOTE BUSTER_TLS_L   tls1      1.61533E+00  1.36273E+00  7.21546E-01  2.44049E-01 -5.13123E-01  3.00651E-01
NOTE BUSTER_TLS_S   tls1      2.26938E-01 -2.36880E-01  2.82462E-01 -1.82387E-01 -6.13010E-02 -2.07300E-01 -1.26450E-02 -1.05032E-01
NOTE BUSTER_TLS_O   tls1     -2.33533E+01 -8.36748E+01  1.13031E+01
NOTE BUSTER_TLS_FIX tls1 ALL
 
NOTE BUSTER_TLS_SET tls2 { B|* }
NOTE BUSTER_TLS_T   tls2     -2.55147E-01 -1.97931E-01  3.33849E-01  1.93375E-02  4.44013E-02 -1.38678E-02
NOTE BUSTER_TLS_L   tls2      1.50139E+00  1.37402E+00  9.53074E-01  7.53471E-01 -8.67385E-01 -7.93362E-01
NOTE BUSTER_TLS_S   tls2     -5.06241E-02  1.57153E-01 -1.23636E-01  1.18337E-01  2.46232E-01  7.25730E-02 -9.77238E-02  6.96725E-02
NOTE BUSTER_TLS_O   tls2      2.20712E+01 -7.19054E+01  3.51200E+00
NOTE BUSTER_TLS_FIX tls2 ALL

 For final position, Geometry function summary
     term                           number     weight       rms        GooF      function     fn/numb
     ================================================================================================
     BOND bond lengths (angs)       5652       2.000      0.0083       0.413      1926.09       0.341
     ANGL bond angles (degs)        7669       2.000        1.03       0.612      5742.98       0.749
     TORS split-harmonic tors       1269       0.000       18.70       1.177         0.00       0.000
     SINTOR sinusoidal tors         1269       2.000       18.70                  2599.70       2.049
     PLAN general plane (angs)       963       5.000      0.0151       0.757      2760.48       2.867
     BCORrelations (angs^2)         5652      10.000       0.984       0.351      6966.75       1.233
     CONT (bad contacts)               0       5.000                                 0.00
     IDEAL(ideal-dist contact)      5532       4.000                             12119.45
     CHIRAL (gelly semiharmon)       740       5.000                                60.73
     SIM similarity restraint for NCS and/or TARGET                               9925.56
     OCCSUM restraint on sum of occupanies                                           0.00       as none defined
     DISTAN utility restraints interatomic distance                                  0.00       as none defined
     UTILANGLE utility angle restraints                                              0.00       as none defined
     UTILTOR utility restraints on torsion angles                                    0.00       as none defined
     Peptide omega torsion angles                           3.06 degs - monitor only

 Weighted rms      0.0083         1.027  (temporary rms bond and angle for BUSTER to pick up!)
 Weighted intra-residual rms(bond) for StdProtein (excluding H)      0.0081
 Weighted intra-residual rms(bond) for Buffer     (excluding H)      0.0031
 Outputing geometry progress graph in plotmtv format to file:Geom_Res.mtv

 ############### Running final geometry/screen analysis (screen_final.txt)

 s/r gelly normal termination
```

Statistics and scale factors vs. cycle number
```

|  |  |  |
| --- | --- | --- |
|  | - Rxpct factors- <Log-Lik Gain>- GEOM Residuals- Ln Sigma_A_ | - Scale Factors- B Factors- Imperfection Factors |

```
   Final Geometry Information


---


### 2. Final results

Statistics and scale factors vs. cycle number
```

|  |  |  |
| --- | --- | --- |
|  | - Rxpct factors- <Log-Lik Gain>- GEOM Residuals- Ln Sigma_A_ | - Scale Factors- B Factors- Imperfection Factors |

```
   Average Figure of Merit
   Structure Factor Amplitudes


 # normal termination

  Program stopped.
```

closesok message: time spent in tree = 1647.67 (user) 33.91 (sys)

---

# Normal termination

```
 Resources usage summary:
     Time spent in client ...................  1647.68 (user)       33.92 (system)
     Time spent in scripts (    27 calls) ...     2.40 (user)        0.17 (system)
     Time spent in server ...................     0.00 (user)        0.01 (system)

     Max resident memory used (kb) .......... 418028
     Number of page faults .................. 0 (major)    373378 (minor)
     Number of context switches ............. 2107479 (niced)    6819 (other)
```
